# Investigation of the In Vivo Metabolism of Sibirioside A and Angoroside C in Rats by HPLC-ESI-IT-TOF-MS^n^

**DOI:** 10.3390/molecules23102702

**Published:** 2018-10-19

**Authors:** Yi-Fan Zhang, Li-Jia Liu, Feng Xu, Ming-Ying Shang, Guang-Xue Liu, Shao-Qing Cai

**Affiliations:** Division of Pharmacognosy, School of Pharmaceutical Sciences, Peking University Health Science Center, No. 38, Xueyuan Road, Beijing 100191, China; zhangyf0911@163.com (Y.-F.Z.); lily199109231@163.com (L.-J.L.); xufeng_pharm@163.com (F.X.); guangxl@bjmu.edu.cn (G.-X.L.); sqcai@bjmu.edu.cn (S.-Q.C.)

**Keywords:** sibirioside A, angoroside C, metabolism, HPLC-IT-TOF-MS^n^, Scrophulariae Radix

## Abstract

Sibirioside A and angoroside C are two important phenylpropanoid glycosides of the traditional Chinese medicine Scrophulariae Radix. High performance liquid chromatography, coupled with an ion trap time-of-flight multistage mass spectrometry equipped with electrospray ionization source (HPLC-ESI-IT-TOF-MS^n^), was applied to the profile and we identified the metabolites of sibirioside A and angoroside C in vivo in rats. A total of four metabolites of sibirioside A were identified: SM1, SM2 and SM3 which were known as new compounds. A total of 25 metabolites were detected for angoroside C: AM4, AM5, AM6, AM7, AM16, AM17, AM20, AM21, AM22, AM23 and AM25 which were identified to be new compounds. The main metabolic reactions were hydrolysis, reduction, hydroxylation, methylation, sulfation, and gluconylation. The prototype of sibirioside A was widely distributed in tissues found in the heart, liver, spleen, lung, kidney, stomach and small intestine of rats, and mainly distributed in the stomach, small intestine, kidney and liver. But for angoroside C, nothing was found in the viscera except the stomach and small intestine. The metabolites of sibirioside A were mainly eliminated from feces, while it was urine for the metabolites of angoroside C. Furthermore, 19 metabolites were likely to have bioactivities based on the ‘PharmMapper’ analysis, which roughly matched the known pharmacological activities of Scrophulariae Radix (SR) and the prototypes. One of the main pharmacological activities of SR in traditional Chinese medicine is anti-diabetes, and the predicted results showed that SM1, SM2, SM3, AM2, AM4, AM5, AM6, AM9, AM10, AM11, AM12, AM13, AM15, AM18, AM19, AM24, and AM25 might be used to cure diabetes. These findings provide a reference for studying the metabolism, distribution and pharmacological actions of phenylpropanoid glycosides in vivo.

## 1. Introduction

Phenylpropanoid glycosides are the most abundant and important constituents, secondary to iridoid glycosides in Scrophulariae Radix (SR), and the representative components include acteoside, angoroside C, sibirioside A and so on [1]. Some research reported that angoroside C can take many pharmacological roles, including anti-inflammatory [2,3,4], antioxidation [5], and cardiovascular protection [6]. For sibirioside A, there was paucity of reports on its pharmacological activity, but only a few literatures about isolation and purification [7,8,9]. SR is widely used in traditional Chinese medicine, derived from the dried roots of *Scrophularia ningpoensis* Hemsl. [10]. SR exhibits a variety of pharmacological actions such as anti-inflammation, analgesia, immunoregulation, blood sugar regulation, anticoagulation, cardiovascular protection, and neuroprotection [11,12,13,14,15]. The pharmacopoeia stipulates that the traditional processing method for SR is “sweating-drying” [10], and previous research indicated that the content of angoroside C decreased after traditional processes while sibirioside A was the opposite [16]. It is a novel and promising way to have some insight into the mechanism of crude drug processing in metabolic terms. The rudimentary metabolic characteristics and pathways of harpagoside [17] and harpagide [18] have been resolved. To investigate the in vivo metabolic process of phenylpropanoid glycosides, we chose sibirioside A and angoroside C as research objects.

To explore the metabolism and distribution of compounds in biological samples, high performance liquid chromatography coupled with multi-stage mass spectrometry (HPLC-MS^n^) is known as an advantageous method, as it provides sensitive, high-resolution and accurate information on hundreds of compounds simultaneously [17]. In this study, we applied the HPLC-IT-TOF-MS^n^ method to investigate the metabolism of sibirioside A and angoroside C and the distribution of their metabolites in rats.

## 2. Results

### 2.1. Fragmentation Behaviors of Sibirioside A and Angoroside C

**Figure 1:**
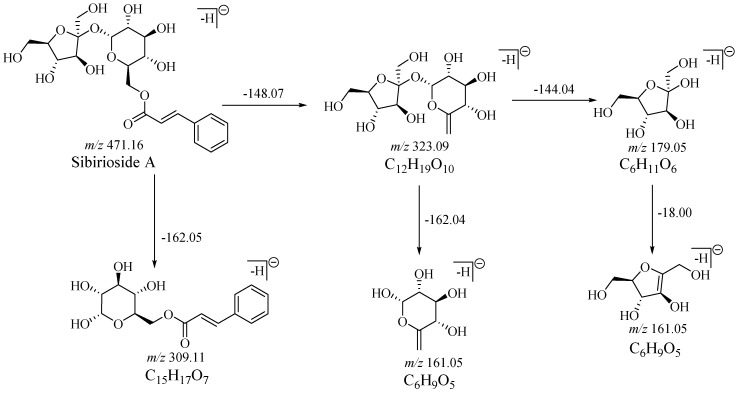
The fragmentation processes of sibirioside A.

**Figure 2:**
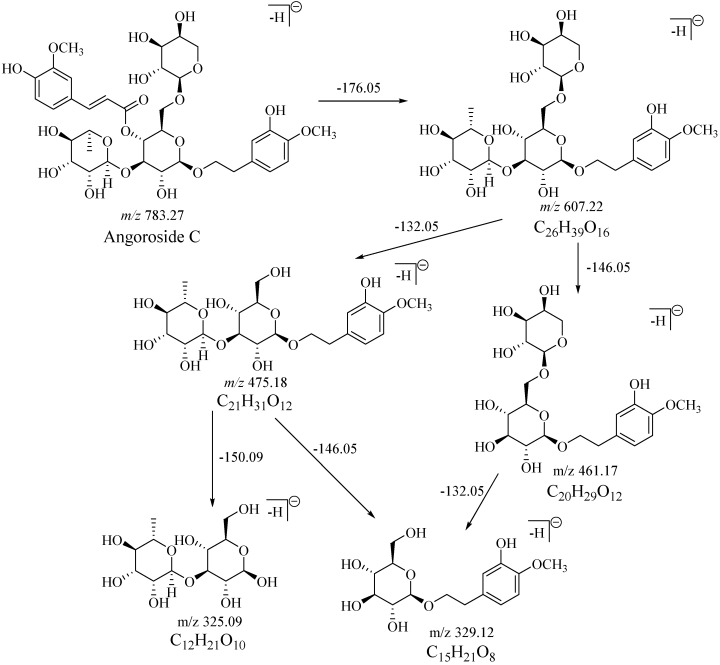
The fragmentation processes of angoroside C.

Aqueous solutions of the reference substances, sibirioside A and angoroside C, were directly injected for analysis to obtain the fragmentation characteristics of the parent compounds. Several characteristic fragments of sibirioside A and angoroside C were observed (Figure S1) in the negative ion mode mass spectrum. Sibirioside A showed quasimolecular ion peaks at *m*/*z* 517.1450 ([M + HCOO]^−^) and *m*/*z* 471.1414 ([M − H]^−^). Its MS^2^ spectra fragments *m*/*z* 323.0918 ([M-cinnamic acid − H]^−^), *m*/*z* 309.0991 ([M − fructosyl − H]^−^), *m*/*z* 179.0523 ([glucose − H]^−^ or [fructose − H]^−^), *m*/*z* 161.0401 ([glucose − H_2_O − H]^−^ or [fructose − H_2_O − H]^−^). The quasimolecular ion peak of angoroside C was *m*/*z* 783.2513 ([M − H]^−^), and the MS^2^ fragments were shown as *m*/*z* 607.1993, *m*/*z* 475.1826, *m*/*z* 461.1514, *m*/*z* 443.1411, *m*/*z* 329.1078, *m*/*z* 325.0911. The fragmentation processes of sibirioside A and angoroside C were shown in Figure 1 and Figure 2.

In total, four sibirioside A metabolites and 25 angoroside C metabolites were identified, and the Liquid Chromatography Mass Spectrometry (LC-MS) results are shown in Table 1.

### 2.2. Metabolites of Sibirioside A: ***SM1**–**SM4***

The negative quasi-molecular ion of **SM1** at *m*/*z* 229.0153 ([M − H]^−^) was detected in feces and urine samples, and speculated as C_9_H_10_O_5_S. Its degrees of unsaturation were five, meaning there was only one unsaturated bond except for the phenyl group in the molecule. The MS^2^ spectrum showed fragment at *m*/*z* 147.0459 (speculated to be [C_9_H_8_O_2_ − H]^−^), and its MS^3^ fragment ion at *m*/*z* 103.0348 ([C_9_H_8_O_2_ − CO_2_ − H]^−^) indicated that C_9_H_8_O_2_ might be cinnamic acid. Cinnamic acid was the product of SM1 with 82 Da lost, which was considered to be H_2_SO_3_. It is well-known that the sulphated compounds could easily lose 80 Da radical group (SO_3_) instead of 82 Da, but the neutral molecule H_2_SO_3_ might be lost if there was active-H in the phenylpropionic acid structure. Then we could conjecture **SM1** as phenylpropionic acid sulphate. The cinnamic acid first reacted with glutathione and then hydrolyzed into the cysteine adduct by peptidase, and the cysteine β-liase gave a thiol immediately after, and finally oxidized to be **SM1**.

**SM2** detected in feces samples showed quasi-molecular ion peak at *m*/*z* 259.0293 ([M − H]^−^), indicating C_10_H_12_O_6_S as the predicted formula. The characteristic fragment ion peaks were shown as *m*/*z* 177.0540 ([M − H_2_SO_3_ − H]^−^) and *m*/*z* 133.0983 ([M − H_2_SO_3_ − CO_2_ − H]^−^). Its trends of neutral loss conformed with those of SM1, and all the fragment ion peaks were 30 Da (predicted to be CH_2_O) larger than the correspondent peaks of SM1, which suggested the substitution of methoxyl group. The methoxyl group was the best possibility to substitute at the aromatic ring owing to the absence of methyl (14 Da) or methoxyl (30 Da) loss in MS^2^ spectrum. Then it could be speculated that **SM2** might be 4-methoxyphenylpropanoic acid sulphate in consideration of the substitutive resistance.

The different chromatographic peak of **SM3** was observed in feces samples, and showed quasi-molecular ion peak at *m*/*z* 393.0755 ([M − H]^−^). The fragment ion at *m*/*z* 311.1019 ([M − H_2_SO_3_ − H]^−^) suggested SM3 to be a sulphate, and it could be dissociated to get *m*/*z* 163.0583 and *m*/*z* 147.0454. There was no fragment ion at *m*/*z* 103.03 rooted from *m*/*z* 147.0454, which indicated that the ion at *m*/*z* 147.0454 was not cinnamic acid. According to the elemental composition C_9_H_8_O_2_, this fragment was most likely to be β-oxobenzenepropanal. In accordance with the above analysis, **SM3** might be β-hydroxybenzenepropanoic anhydride sulphate.

The molecular formula of **SM4** detected in stomach samples was predicted to be C_27_H_38_O_17_ according to the quasi-molecular ion peak at *m*/*z* 633.2014 ([M − H]^−^). Its MS^2^ spectrum showed fragment ion peaks at *m*/*z* 485.1416 ([M − cinnamoyl − H]^−^) and *m*/*z* 323.0932 ([sucrose − H]^−^). Neutral loss of 148 Da (cinnamic acid) occurred from the parent ion to obtain *m*/*z* 485.1416, then neutral loss of 162 Da (glucosyl) occurred from *m*/*z* 485.1416 to get *m*/*z* 323.0932, which was the main fragment ion of sibirioside A predicted to be [sucrose − H]^−^. To sum up, **SM4** was conjectured to be a sibirioside A glucose conjugation.

**Figure 3:**
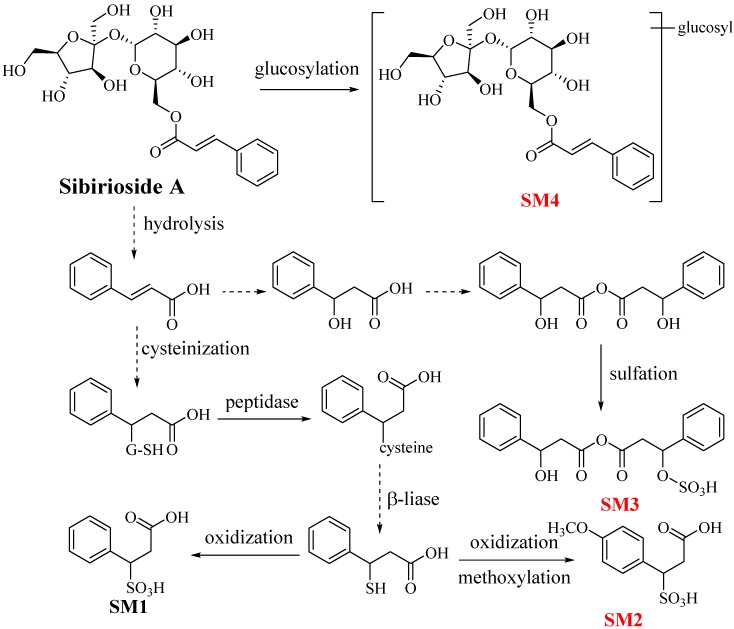
The metabolites and metabolic pathways of sibirioside A in rats.

The metabolites and metabolic pathways of sibirioside A in rats are shown in Figure 3.

### 2.3. Metabolites of Angoroside C: ***AM1**–**AM25***

**AM1** showed [M − H]^−^ ion peak at *m*/*z* 233.0119 ([M − H]^−^) in the negative MS^1^ spectrum, which was predicted as C_8_H_10_O_6_S. The fragment ion peaks at *m*/*z* 153.0583 ([M − SO_3_ − H]^−^) and *m*/*z* 123.0492 ([M − SO_3_ − CH_2_O − H]^−^) were observed in negative MS^2^ spectrum, and *m*/*z* 153.0583 should be the phenylethanoid unit derived from angoroside C. Therefore, **AM1** was speculated to be 4-(2-hydroxyethyl)-1,2-benzenediol sulphate.

The formula of **AM2** was determined to be C_9_H_8_O_6_S based on its quasi-molecular ion peak at *m*/*z* 242.9965 ([M − H]^−^). The characteristic fragment ion peaks were observed as *m*/*z* 163.0415 ([M − SO_3_ − H]^−^) and *m*/*z* 119.0532 ([M − SO_3_ − CO_2_ − H]^−^). Therefore, the ion at *m*/*z* 163.0415 was thought to be the demethoxylation product of ferulic acid, which was the hydrolysate of angoroside C. And **AM2** was thus determined as *p*-coumaric acid sulphate. It was easy to confirm that **AM3** was 4-hydroxybenzenepropanoic acid sulphate, the hydrogenation product of **AM2** based on the quasi-molecular ion peak at *m*/*z* 245.0109 ([M − H]^−^) and its MS^2^ fragment ion peaks at *m*/*z* 165.0574 ([M − SO_3_ − H]^−^) and *m*/*z* 121.0680 ([M − SO_3_ − CO_2_ − H]^−^).

For **AM4**, **AM5** and **AM6**, the exact formula C_9_H_12_O_6_S was calculated based on the quasi-molecular ion peak at *m*/*z* 247.03 ([M − H]^−^). The ion at *m*/*z* 167.07 ([M − SO_3_ − H]^−^) was detected in MS^2^ spectrum, which suggested that they were 3,4-hydroxyphenylpropanol sulphate. And there were three reaction sites in 3,4-hydroxyphenylpropanol for sulphating, so the three metabolites were considered as isomers.

The quasi-molecular ion peak of **AM7** at *m*/*z* 259.0271 ([M − H]^−^) suggested the formula as C_10_H_12_O_6_S. And the fragment ion peaks at *m*/*z* 179.0726 ([M − SO_3_ − H]^−^), *m*/*z* 163.0385 ([M − SO_3_ − CH_2_ − H]^−^), *m*/*z* 147.0473 ([M − SO_3_ − CH_4_O − H]^−^, namely [cinnamic acid − H]^−^) and *m*/*z* 119.0507 ([M − SO_3_ − CH_2_ − CO_2_ − H]^−^) indicated the metabolite might be methoxybenzenepropanoic acid sulphate, which was a isomer of **SM2**.

**AM8** and **AM9** were speculated to be C_9_H_10_O_7_S owing to the quasi-molecular ion peak at *m*/*z* 261.01 ([M − H]^−^) in negative ion mode MS^1^ spectrum. The multistage fragment ion peaks at *m*/*z* 217.0175 ([M − CO_2_ − H]^−^), *m*/*z* 181.0522 ([M − SO_3_ − H]^−^) and *m*/*z* 137.0633 ([M − SO_3_ − CO_2_ − H]^−^) indicated that they were 3,4-dihydroxybenzenepropanoic acid-*O*-sulphate.

The quasi-molecular ion peak at *m*/*z* 275.02 ([M − H]^−^) observed in the negative MS^1^ spectrum indicated the formula C_10_H_12_O_7_S. Then, it was speculated that **AM10** was 3-methoxyl-4-hydroxybenzenepropanoic acid sulphate and that **AM11** was isomer of it owing to the fragment ion peaks at *m*/*z* 195.0665 ([M − SO_3_ − H]^−^), *m*/*z* 151.0799 ([M − SO_3_ − CO_2_ − H]^−^) and *m*/*z* 177.0777 ([M − SO_3_ − H_2_O − H]^−^) in the MS^n^ spectrum.

The [M − H]^−^ peaks of **AM12**, **AM13** and **AM14** were observed at *m*/*z* 329.09, suggesting the formula as C_14_H_18_O_9_. The parent ion was cleaved into two parts *m*/*z* 175.0282 ([glucuronic acid − H_2_O − H]^−^) and *m*/*z* 153.0568. And the ion at *m*/*z* 153.0568 was determined to be 3,4-dihydroxyphenylethanol similar to **AM1**, which has three reaction sites for gluconylation. The above data suggested **AM12**, **AM13** and **AM14** to be 4-(2-hydroxyethyl)-1,2-benzenediol glucuronide.

**AM15,** speculated to be C_15_H_18_O_9,_ was detected in urine samples with quasi-molecular ion peak at *m*/*z* 341.0848 ([M − H]^−^). The MS^2^ fragment ion peaks at *m*/*z* 165.0520 ([M − glucuronic acid − H]^−^) and *m*/*z* 121.0628 ([M − glucuronic acid − CO_2_ − H]^−^) were just similar to **AM3**, and in combination with *m*/*z* 175.0244, **AM15** was determined to be 4-hydroxybenzenepropanoic acid glucuronide. And the MS^1^ chromatographic peaks of **AM16** and **AM17** were extracted in urine sample at *m*/*z* 343.1017 ([M − H]^−^). Their fragment ion peaks at *m*/*z* 175.0265 and *m*/*z* 167.0713 indicated that they were 3,4-hydroxyphenylpropanol glucuronide, the reduzates of **AM15**.

The [M − H]^−^ peak of **AM18** at *m*/*z* 369.0794 (C_16_H_18_O_10_) was detected in urine samples. The MS^2^ fragment ion at *m*/*z* 175.0169 suggested the existence of glucuronic acid, and *m*/*z* 193.0508 (C_9_H_10_O_4_) was consistent with the phenylpropanoid unit of angoroside C. Therefore, **AM18** might be 3-methoxyl-4-hydroxycinnamic acid glucuronide. And the quasi-molecular ion peak at *m*/*z* 371.0988 ([M − H]^−^) and the MS^2^ fragment ion peaks at *m*/*z* 175.0265 and *m*/*z* 195.0658 of **AM19** suggested that it could be the hydrogenation product of **AM18**, namely 3-methoxyl-4-hydroxybenzenepropanoic acid glucuronide.

The ion peaks at *m*/*z* 637.21 were the quasi-molecular ions of **AM20** and **AM21**, which was the characteristic fragment ion of angoroside C. And the MS^2^ fragment ion peaks at *m*/*z* 461.1636, *m*/*z* 311.1151, *m*/*z* 193.0548, *m*/*z* 167.1269 and *m*/*z* 137.0759 were also in conformity with angoroside C. Then they might be the isomers of deglycosylated angoroside C. For **AM22** and **AM23**, the quasi-molecular ion peaks at *m*/*z* 959.30 and main MS^2^ fragment ion peak at *m*/*z* 783.26 ([angoroside C − H]^−^) were observed, which indicated **AM22** and **AM23** to be isomers of angoroside C glucuronide.

The formula of **AM24** detected in feces samples was predicted to be C_20_H_30_O_12_ based on the quasi-molecular ion peak at *m*/*z* 461.1701. The fragment ion peaks at *m*/*z* 443.1711 and *m*/*z* 329.1180 could also be observed in the MS^2^ spectrum of angoroside C. So it could be speculated that **AM24** was the hydrolysate of deferuloyl-angoroside C, namely 2-(3-hydroxy-4-methoxyphenyl)ethyl 6-*O*-arabinopyranosyl glucopyranoside.

The negative quasi-molecular ion of **AM25** at *m*/*z* 247.0266 ([M − H]^−^) was detected in feces and plasma samples, and speculated as C_9_H_12_O_6_S. The fragment ion peaks at *m*/*z* 167.0750 ([M − SO_3_ − H]^−^), *m*/*z* 137.0627 ([M − SO_3_ − CH_2_O − H]^−^) and *m*/*z* 121.0264 ([M − SO_3_ − CH_2_O − CH_4_ − H]^−^) suggested that **AM25** was 4-(2-hydroxyethyl)-1,2-benzenediol sulphate.

**Figure 4:**
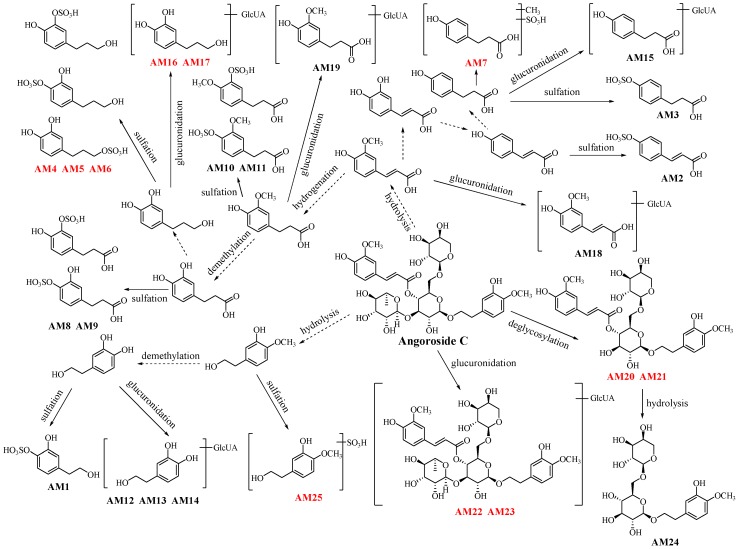
The metabolites and metabolic pathways of angoroside C in rats.

The metabolites and metabolic pathways of angoroside C in rats are shown in Figure 4.

### 2.4. The Distribution of Sibirioside A and Angoroside C in Rats

**Figure 5:**
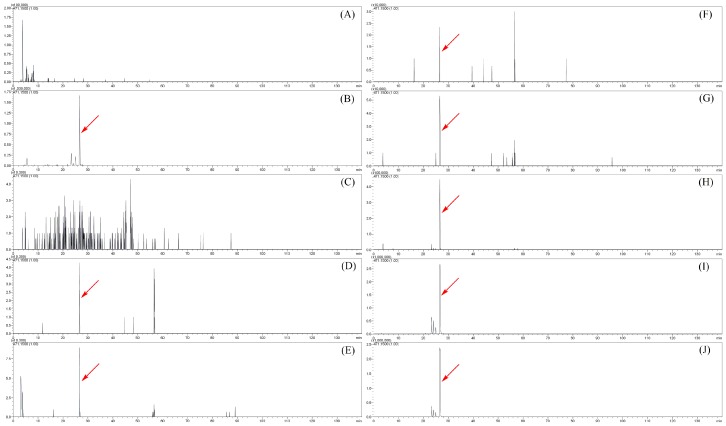
The extract ion flow chromatography (EIC) at *m*/*z* 471.1500 of sibirioside A in different biological samples. (**A**) plasma; (**B**) urine; (**C**) feces; (**D**) heart; (**E**) liver; (**F**) spleen; (**G**) lung; (**H**) kidney; (**I**) stomach; (**J**) small intestine.

**Figure 6:**
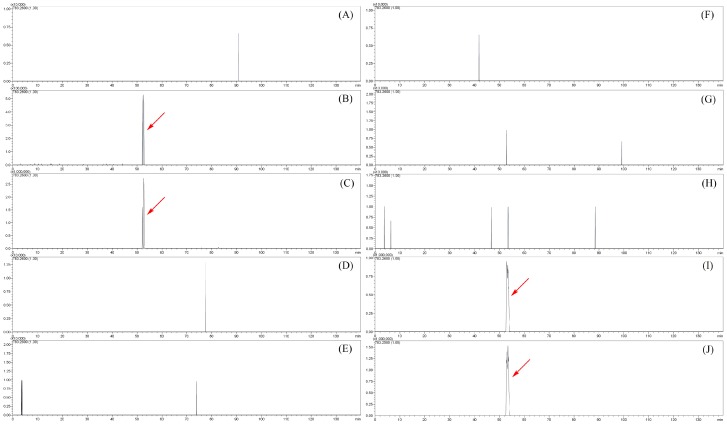
The EIC at *m*/*z* 783.2600 of angoroside C in different biological samples. (**A**) plasma; (**B**) urine; (**C**) feces; (**D**) heart; (**E**) liver; (**F**) spleen; (**G**) lung; (**H**) kidney; (**I**) stomach; (**J**) small intestine.

To investigate the distribution of the prototypes of sibirioside A and angoroside C, the corresponding quasi-molecular ion peaks at *m*/*z* 471.14 and *m*/*z* 783.25 were extracted in negative MS^1^ spectrum. The extract ion flow chromatography (EIC) of different biological samples are shown in Figure 5 and Figure 6. The prototype of sibirioside A was widely distributed in rats; it could be detected in urine, heart, liver, spleen, lung, kidney, stomach, and small intestine samples, and the content of sibirioside A in the stomach and small intestine is the highest, followed by the kidney and liver. Angoroside C was detected in urine, feces, stomach, and small intestine samples, while there was only one metabolite observed in plasma samples without the prototype of angoroside C.

### 2.5. Biological Activity Prediction of the Metabolites of Sibirioside A and Angoroside C

The bio-activities of the metabolites of sibirioside A and angoroside C were predicted through “PharmMapper Server”, which based on a massive pharmacophore database containing relatively well-developed target information over 7000 pharmacophore models recorded in TargetBank, BindingDB, DrugBank, and other drug target databases [30,31,32]. It automatically matches the pending molecules with all targets in the database and then calculates the fit degree to list the best scoring targets. The first 50 results on human targets were chosen and then the disease-related outcomes for every metabolite were picked out. These are shown in Table S1. In total, 29 targets were picked out, of which RASH, INSR, KPYR, ANGI, BTK, FA7, NDKB, G6PI and CAH2 were identified over 10 times. Their functions include anti-cancer, anti-diabetes, anti-anemia, neuroprotection, anti-leukemia, anti-coagulopathy, and osteogenic activity. The most common target is RASH, which has GTPase activity and is considered to be related to inhibition of growth and metastasis of tumors. Defects in INSR, an insulin binding receptor with tyrosine kinase activity, could cause insulin resistance, leprechaunism, noninsulin-dependent diabetes mellitus, and familial hyperinsulinemic hypoglycemia. This target was identified as a potential target of 17 metabolites of sibirioside A and angoroside C. Defects in INSR cause insulin resistance, leprechaunism, noninsulin-dependent diabetes mellitus, and familial hyperinsulinemic hypoglycemia. Thus, **SM1**, **SM2**, **SM3**, **AM2**, **AM4**, **AM5**, **AM6**, **AM9**, **AM10**, **AM11**, **AM12**, **AM13**, **AM15**, **AM18**, **AM19**, **AM24**, **AM25** might be used to cure diabetes, which was one of the main pharmacological activities of SR in traditional Chinese medicine. In addition, **SM3**, **AM2**, **AM5**, **AM8**, **AM9**, **AM10**, **AM11**, **AM14**, **AM15**, **AM18**, **AM19**, **AM24** and **AM25** may be available to target chronic leukemia; **AM2**, **AM4**, **AM5**, **AM6**, **AM8**, **AM9**, **AM10**, **AM11**, **AM14** and **AM24** may have osteogenic actions. The predicted outcomes were in good agreement with the actual activities reported in the literatures [11,12,13,14,15].

The metabolites of sibirioside A and angoroside C have a variety of potential pharmacological actions, which require further research.

## 3. Discussion

A total of 4 metabolites of sibirioside A were found in biological samples, of which **SM1** was in urine, **SM1**–**SM3** in feces, and SM4 in the stomach. A total of 25 metabolites of angoroside C were found and identified, of which **AM1**–**AM23** were in urine, **AM24** and **AM25** were in feces, and AM25 in plasma. For sibirioside A, the main metabolic reactions were hydrogenation, hydroxylation, methylation, sulfation, glycosylation, and dimerization, while the metabolic reaction of angoroside C included hydrolyzation, hydrogenation, sulfation, gluconylation, demethylation, and dehydroxylation. The hydrolyzation reactions occurred in the sugar ester bonds, while the hydrogenation reactions were mainly in double bonds or carboxyl. The sulfation reactive site was the carboxyl group for sibirioside A, but it was the hydroxyl group for angoroside C. The results showed that gluconylation was one of the prime and representative metabolic reactions for angoroside C.

Sibirioside A is an ester glycoside consisting of cinnamic acid and sucrose, and there were less metabolites of it due to the simple molecular structure. The relatively large polarity allowed it to be easily excreted from the urine without the need for complicated metabolic processes. Catalyzed by a variety of enzymes, a portion of sibirioside A was transformed into the metabolites which were mainly sulfated products, then discharged from feces through the bile. The prototype of sibirioside A was detected in all the selected samples except for feces and plasma, which indicated that the rate of sibirioside A clearance was relatively fast in the blood, and it was mainly distributed in the heart, liver, spleen, kidney, stomach, and small intestine in the form of prototype. It was conjectured that sibirioside A might work in the original form and could be preserved for a long time in the viscera in order to extend the time for drug concentration.

Angoroside C is a phenylpropanoid glycoside consisting of three monosaccharide, a phenylpropanoid unit, and a phenylethanoid unit. For angoroside C, total of 25 metabolites were identified, much more than that of sibirioside A, of which 22 were sulfate and glucuronide conjugates. The prototype of angoroside C was detected in urine, feces, stomach, and small intestine samples, but not observed in plasma. It was speculated that the clearance rate of angoroside C in plasma might be fast like sibirioside A. A total of 22 metabolites derived from urine samples had covered all types of metabolic reactions found in this study, and most of the prototype and metabolites were rapidly excreted from the urine.

## 4. Materials and Methods

### 4.1. Chemicals and Materials

Sibirioside A (>98%, Lot MUST-16121505) and angoroside C (99%, Lot MUST-16012611) were purchased from Chendu Must Bio-Technology Co., Ltd. (Chengdu, China). Acetonitrile (Fisher, Pittsburgh, PA, USA) and formic acid (ROE, Santa Monica, CA, USA) were of HPLC-grade, and ultrapure water was prepared by Milli-Q water purification system (Millipore, Billerica, MA, USA).

### 4.2. Animal Experiments

Nine male Sprague-Dawley rats weighing around 250 g were obtained from the Experimental Animal Center of Peking University’s Health Science Center (Beijing, China). The rats were housed in metabolic cages (type DXL-DL, Suzhou Fengshi Laboratory Animal Equipment Co. Ltd., Suzhou, China) kept in an environmentally controlled breeding room for one week for acclimation and had unrestricted access to laboratory-made chow and distilled water to reduce the biological background interferences. Nine rats were divided into three groups, namely three rats in each group (group A, blank group; group B, sibirioside A group; group C, angoroside C group). The standards of sibirioside A and angoroside C were dissolved in ultrapure water and orally administrated respectively to group B and group C at the dose of 200 mg/kg for six continuous days. The rats in group A were orally treated with the same volume of water. All animal experiments were conducted in accordance with the Guide for the Care and Use of Laboratory Animals of the US National Institutes of Health. Experiments were reviewed by the Biomedical Ethical Committee of Peking University (approval no. LA2015-007). During the continuous six days of administration, the feces and urine samples were dividually collected using the metabolic cages, and the heparin anticoagulant blood sample was collected by heart puncture under anesthesia 1 h after the last administration. The hearts, livers, spleens, lungs, kidneys and small intestines were quickly scissored and lavaged by normal saline, then the tissue samples were stored at −80 °C.

### 4.3. Preparation of Rat Biological Samples

Blood samples were centrifuged at 5000 rpm (type 3K15, Sigma Laborzentrifugen GmbH, Osterode am Harz, Germany) for 15 min to obtain plasma samples. The plasma samples were precipitated with four times methanol, then centrifuged at 10,000 rpm for 15 min. The supernatant was dried under reduced pressure at room temperature. The residue was redissolved in one-twelfth of methanol and centrifuged at 15,000 rpm for 20 min. The supernatant was collected for analysis.

Urine samples were dried under reduced pressure at 50 °C (Heidolph Laborota 4001 rotatory evaporator, Heidolph Instruments GmbH & Co., Schwabach, Germany); the residue was ultrasonically extracted in 50 mL methanol for 30 min. The suspension was centrifuged at 5000 rpm for 15 min, and the supernatant was dried under reduced pressure at 40 °C. The residue was redissolved in five times of methanol, and filtered through a 0.22 µm nylon filter.

Feces samples were dried in a thermostat oven (Shanghai Yiheng Scientific Instruments Co., Ltd., Shanghai, China) at 50 °C, and then pulverized to get powders. The feces powders were ultrasonically extracted for 30 min in five times of 50% methanol, repeated three times. The extracts were merged and dried under reduced pressure at 40 °C, and the residue was redissolved in 10 times of methanol, then filtered through a 0.22 µm nylon filter.

Organ tissues were homogenized by an ultra-turrax T10 electric homogenizer (IKA Co., Ltd., Staufen, Germany) with twice as much water, and five times of methanol was added into the homogenate. The mixture underwent ultrasonic processing for 30 min, thencentrifuged at 15,000 rpm for 20 min. The supernatant was dried under reduced pressure at room temperature. The residue was redissolved in methanol and centrifuged at 15,000 rpm for 20 min. The supernatant was collected for analysis.

### 4.4. Instrumental and Analytical Conditions

HPLC analysis was performed on a Shimadzu HPLC system equipped with two LC-20AD pumps, a CTO-20A column oven, an SIL-20 AC autosampler, and a CBM-20A system controller. Chromatographic separation was carried out on an Agilent Zorbax SB-C_18_ column (5 μm, 250 × 4.6 mm) protected by an Agilent Zorbax SB-C_18_ column. A 10 μL aliquot was injected for each sample. The thermostatted autosampler was maintained at 15 °C and the column oven temperature was kept at 30 °C. The column was eluted with a gradient mobile phase consisting of 0.1% formic acid aqueous solution (solvent A) and acetonitrile (solvent B) with a flow rate of 1.0 mL/min. For the sibirioside A group, the gradient program was adopted in the following manner: 5–18% B at 0–20 min, 18–37% B at 20–40 min, 37–43% B at 40–43 min, 43–56% B at 43–63 min, 56–68% B at 63–65 min, 68–81% B at 65–75 min, 81–100% B at 75–95 min and 100% B at 95–140 min. For the angoroside C group, the gradient program was adopted in the following manner: 3% B at 0–8 min, 3–5% B at 8–10 min, 5% B at 10–20 min, 5–8% B at 20–23 min, 8–19% B at 23–38 min, 19% B at 38–45 min, 19–27% B at 45–50 min, 27% B at 50–63 min, 27–55% B at 63–73 min, 55% B at 73–83 min, 55–100% B at 83–93 min and 100% B at 93–140 min. High-resolution mass spectra were recorded on an IT-TOF mass spectrometer (Shimadzu, Kyoto, Japan). The ESI source was operated in negative ion mode. The mass spectrometer was programmed to carry out a full scan over *m*/*z* 100–1000 Da (MS_1_) and *m*/*z* 50–1000 Da (MS_2_ and MS_3_). The other parameters were set as follows: flow rate, 0.2 mL/min (split from 1.0 mL/min HPLC effluent); heat block and curved desolvation line temperature, 200 °C; nebulizing nitrogen gas flow, 1.5 L/min; interface voltage: (+), 4.5 kV; (−), −3.5 kV; detector voltage, 1.70 kV; relative collision-induced dissociation energy, 50%. All data were recorded and processed using Shimadzu software LCMS solution version 3.60 and Formula Predictor version 1.2 (Shimadzu, Kyoto, Japan).

## 5. Conclusions

A total of four metabolites of sibirioside A were detected and identified, three of which were new compounds. The main metabolic reactions included hydrogenation, hydroxylation, methylation, sulfation, glycosylation, and dimerization, and the different types of reactions usually occurred simultaneously. Sibirioside A was mainly excreted from urine in the form of prototype. The in vivo metabolism might be carried out in the liver, and then excreted from feces through bile. It was speculated that sibirioside A might take effects in prototype form due to its wide distribution in the heart, liver, spleen, lung, kidney, stomach, and small intestine. There were 25 metabolites identified in vivo for angoroside C, 11 of which were new compounds. The prime metabolic reactions were hydrolyzation, hydrogenation, sulfation, glucuronidation, demethylation, and dihydroxylation. A total of 22 of the metabolites were sulfated or glucuronidization products and mainly excreted from urine just like the prototype, which was concentrically distributing in the stomach and small intestine.

In total, 29 targets were identified including INSR, RASH, KPYR, ANGI and so on, which might be related to anti-diabetes, anti-cancer, anti-anemia, neuroprotection, anti-leukemia, anti-coagulopathy, and osteogenic activities. The predicted outcomes indicated that the metabolites of sibirioside A and angoroside C have a variety of potential pharmacological activities similar to the prototypes, which require further research.

Phenylpropanoid glycosides are a large class of compounds with a simple structure and extensive pharmacological activities. Their metabolic processes in vivo are simple and predominantly they might distribute and take effect in the form of prototypes.

## Figures and Tables

**Table 1:** LC-MS data obtained in the negative ion mode for the identification of the metabolites of sibirioside A and angoroside C in rats.

No.	t_R_/min	Formula	*m*/*z*	Diff	DBE	Fragment	Identification
**SM1**	7.813	C_9_H_10_O_5_S	229.0155	−9.17	5	147.0459, 103.0348	phenylpropanoic acid sulphate [19]
**SM2**	10.328	C_10_H_12_O_6_S	259.0293	4.25	5	177.0540, 133.0983	4-methoxyphenylpropanoic acid sulphate *
**SM3**	7.277	C_18_H_18_O_8_S	393.0755	10.50	10	311.1019, 163.0583, 147.0454	β-hydroxybenzenepropanoic anhydride sulphate *
**SM4**	24.513	C_27_H_38_O_17_	633.2014	−3.47	9	485.1416, 323.0932	sibirioside A glucose conjugation *
**AM1**	15.602	C_8_H_10_O_6_S	233.0119	−2.57	4	153.0583, 123.0492	4-(2-hydroxyethyl)-1,2-benzenediol sulphate [20]
**AM2**	32.993	C_9_H_8_O_6_S	242.9965	−7.82	6	163.0415, 119.0532	*p*-coumaric acid sulphate [21]
**AM3**	29.057	C_9_H_10_O_6_S	245.0109	−6.53	5	165.0574, 121.0680	4-hydroxybenzenepropanoic acid sulphate [22]
**AM4**	17.995	C_9_H_12_O_6_S	247.0265	−6.88	4	167.0745, 137.0642	3,4-hydroxyphenylpropanol sulphate 1 *
**AM5**	20.260	C_9_H_12_O_6_S	247.0271	−4.45	4	167.0745, 137.0642	3,4-hydroxyphenylpropanol sulphate 2 *
**AM6**	22.548	C_9_H_12_O_6_S	248.0282	0	4	167.0745, 137.0642	3,4-hydroxyphenylpropanol sulphate 3 *
**AM7**	42.685	C_10_H_12_O_6_S	259.0271	−4.25	5	179.0726, 163.0385, 147.0473, 119.0507	β-methoxybenzenepropanoic acid sulphate *
**AM8**	18.630	C_9_H_10_O_7_S	261.0063	−4.21	5	217.0175, 181.0522, 137.0633	3,4-dihydroxybenzenepropanoic acid-3-*O*-sulphate [23]
**AM9**	26.887	C_9_H_10_O_7_S	261.0051	−8.81	5	217.0175, 181.0522, 137.0633	3,4-dihydroxybenzenepropanoic acid-3-*O*-sulphate [24]
**AM10**	30.600	C_10_H_12_O_7_S	275.0210	−7.64	5	195.0665, 177.0545, 151.0799	3-methoxyl-4-hydroxybenzenepropanoic acid sulphate [25]
**AM11**	38.278	C_10_H_12_O_7_S	275.0214	−6.18	5	195.0665, 177.0545, 151.0799	3-hydroxyl-4- methoxyl benzenepropanoic acid sulphate [24]
**AM12**	15.712	C_14_H_18_O_9_	329.0929	5.1	6	175.0282, 153.0568	4-(2-hydroxyethyl)-1,2-benzenediol glucuronide [26]
**AM13**	16.507	C_14_H_18_O_9_	329.0871	−2.13	6	175.0282, 153.0568	4-(2-hydroxyethyl)-1,2-benzenediol glucuronide [27]
**AM14**	17.792	C_14_H_18_O_9_	329.0857	−6.38	6	175.0282, 153.0568	4-(2-hydroxyethyl)-1,2-benzenediol glucuronide [20]
**AM15**	33.103	C_15_H_18_O_9_	341.0848	−8.80	7	323.0709, 175.0244, 165.052, 121.0628	4-hydroxybenzenepropanoic acid glucuronide [28]
**AM16**	24.020	C_15_H_20_O_9_	343.1017	−5.25	6	325.097, 175.0265, 167.0713, 147.0301	3,4-hydroxyphenylpropanol glucuronide *
**AM17**	29.170	C_15_H_20_O_9_	343.1017	−5.25	6	325.097, 175.0265, 167.0713, 147.0301	3,4-hydroxyphenylpropanol glucuronide *
**AM18**	35.262	C_16_H_18_O_10_	369.0794	−8.94	8	193.0508, 175.0169	3-methoxyl-4-hydroxycinnamic acid glucuronide [25]
**AM19**	32.448	C_16_H_20_O_10_	371.0988	1.08	7	195.0658, 175.0265	3-methoxyl-4-hydroxybenzenepropanoic acid glucuronide [25]
**AM20**	52.445	C_30_H_38_O_15_	637.2131	−1.10	12	461.1636, 443.1533, 311.1151, 275.0889, 193.0548, 167.1269, 137.0759	deglycosylated angoroside C 1 *
**AM21**	53.108	C_30_H_38_O_15_	637.2177	6.12	12	461.1636, 443.1533, 311.1151, 275.0889, 193.0548, 167.1269, 137.0759	deglycosylated angoroside C 2 *
**AM22**	44.933	C_42_H_56_O_25_	959.3007	−3.23	15	783.2594, 607.2227, 461.1681, 443.1608	angoroside C glucuronide 1 *
**AM23**	47.378	C_42_H_56_O_25_	959.2941	−0.31	15	783.2594, 607.2227, 461.1681, 443.1608	angoroside C glucuronide 2 *
**AM24**	33.343	C_20_H_30_O_12_	461.1701	7.81	6	461.1701, 443.1711, 329.1180, 191.0574	2-(3-hydroxy-4-methoxyphenyl)ethyl 6-*O*-arabinopyranosyl glucopyranoside [29]
**AM25**	27.377	C_9_H_12_O_6_S	247.0266	−6.48	4	167.0750, 137.0627, 121.0264	4-(2-hydroxyethyl)-1,2-benzenediol sulphate *

* means new compound.

## Supplementary Materials

Supplementary materials are available online. Figure S1: The ESI-IT-TOF MSn spectra of angoroside C (A) and sibirioside A (B), Figures S2–S20: The negative ion mode EIC, Table S1: The predicted results of bioactivities for metabolites of sibirioside A and angoroside C.

## References

[1] Xu F.Q., Xu X.D., Chen S.L. (2013). Progress in chemical constituents and bioactivities of *Scrophularia ningpoensis*. Mod. Chin. Med..

[2] Li Y.M., Zeng H.W., He X., Jiang Y.Y., Jiang S.H., Zhu D.Y. (1999). Iridoid and phenylpropanoid glycosides of *Scrophularia ningpoensis* inhibit the formation of LTB_4_ and platelet aggregation. Acad. J. Second Mil. Med. Univ..

[3] Díaz A.M., Abad M.J., Femández L., Silván A.M., Santos J.D., Bemejo P. (2004). Phenylpropanoid glycosides from *Scrophularia scorodonia*: In vitro anti-inflammatory activity. Life Sci..

[4] Liu H., Zheng Y.F., Li C.Y., Zheng Y.Y., Wang D.Q., Wu Z., Huang L., Wang Y.G., Li P.B., Peng W. (2015). Discovery of Anti-inflammatory Ingredients in Chinese Herbal Formula Kouyanqing Granule based on Relevance Analysis between Chemical Characters and Biological Effects. Sci. Rep..

[5] Li Y.M., Han Z.H., Jiang S.H., Yao S.D., Zhu D.Y. (2000). Fast repairing of oxidized OH radical adducts of dAMP and dGMP by phenylpropanoid glycosides from *Scrophularia ningpoensis*. Acta Pharmacol. Sin..

[6] Gu W.L., Chen C.X., Huang X.Y., Gao J.P. (2015). The effect of angoroside C on pressure overload-induced ventricular remodeling in rats. Phytomedicine.

[7] Li Y.M., Jiang S.H., Gao W.Y., Zhu D.Y. (2000). Phenylpropanoid glycosides from *Scrophularia ningpoensis*. Phytochemistry.

[8] Jing J., Chan C.O., Xu L., Jin D., Cao X., Mok D.K., Parekh H.S., Chen S. (2011). Development of an in-line HPLC fingerprint ion-trap mass spectrometric method for identification and quality control of Radix Scrophulariae. J. Pharm. Biomed. Anal..

[9] Zhang J., Fanny C.F., Liang Y., Nancy Y., Zhong B.L., Lai C.W., Xu S.H. (2017). A new iridiod glycoside and a new cinnamoyl glycoside from *Scrophularia ningpoensis* Hemsl. Nat. Prod. Res..

[10] Chinese Pharmacopoeia Commission (2015). Pharmacopoeia of the Peoples Republic of China.

[11] Zhang L., Zhang L., Zhang Y.D. (2014). Comparative study on the pharmacognosy and anti-inflammatory activities of *Scrophularia buergeriana* Miq. Var. *tsinglingensis Tsoong* and *S*. *ninpoensis* Hemsl. Northwest Pharm. J..

[12] Bermejo B.P., Diaz L.A., Silvan S.A., De Santos G.Z., Fernandez M.L., Sanz G.A., Abad M.J. (2000). Effects of some iridoids from plant origin on arachidonic acid metabolism in cellular systems. Planta Med..

[13] Liu G.L., Fu P.Y., Wang Z.Y., Xing D.Y. (1991). Effects of water extract of four Chinese herbal drugs on the binding of insulin with human erythroeyte insulin receptor. Chin. J. Integr. Trad. West. Med..

[14] Ni Z., Cai X.Z., Huang Y.P., Wang D.J., Bian H.M. (2004). Effect of extracts of *Scrophularia ningpoensis* Hemsl. on hemorrheology, coagulation and fibrinolysis in rats. J. Chin. Microcirc..

[15] Sohn S., Ko E., Jeon S., Lee B.J., Kim S.H., Dong M.S., Lee D.U., Kwak J.H., Kim Y.S., Shin M.K. (2009). The genome-wide expression profile of *Scrophularia ningpoensis*-treated thapsigargin-stimulated U-87MG cells. Neurotoxicology.

[16] Wang J.Z., Liu Z., Ma L.M., Li J., Shang M.Y., Liu G.X., Xu F., Cai S.Q. (2016). Changes of chemical constituents in Scrophulariae Radix during processing based on UPLC-Q-TOF MS. J. Chin. Mass Spectrom. Soc..

[17] Wang J.Z., Zhang Y.F., Xu F., Shang M.Y., Liu G.X., Cai S.Q. (2018). Investigation of the in vivo metabolism of harpagoside and distribution of its metabolites in rats by HPLC-IT-TOF-MS^n^. Biomed. Chromatogr..

[18] Liu Z., Xu F., Wang J.Z., Liu G.X., Shang M.Y., Cai S.Q. (2017). Identification and analysis of harpagide metabolites in rats in vivo. Chin. Pharm..

[19] Ounnas F., Privé F., Salen P., Gaci N., Tottey W., Calani L., Bresciani L., Lopez-Gutierrez N., Hazane-Puch F., Laporte F. (2016). Whole rye consumption lmproves blood and liver n-3 fatty acid profile and gut microbiota composition in rats. PLoS ONE.

[20] Tuck K.L., Hayball P.J., Stupans I. (2002). Structural characterization of the metabolites of hydroxytyrosol, the principal phenolic component in olive oil, in rats. J. Agric. Food Chem..

[21] Zaklo D., Costagliola R., Dorio C., Rathahao E., Cravedi J.P. (2003). In vivo metabolic fate of the xeno-estrogen 4-*N*-nonylphenol in Wistar rats. Drug Metab. Dispos..

[22] Wusteman F.S., Dodgson K.S., Lloyd A.G., Rose F.A., Tudball N. (1964). Thin-layer chromatography in the study of ester sulfates. J. Chromatogr. A.

[23] Stalmach A., Mullen W.D. (2009). Metabolite profiling of hydroxycinnamate derivatives in plasma and urine after the ingestion of coffee by humans: Identification of biomarkers of coffee consumption. Drug Metab. Dispos..

[24] Fumeaux R., Menozzi C., Stalmach A., Munari C., Kraehenbuehl K. (2010). First synthesis, characterization, and evidence for the presence of hydroxycinnamic acid sulfate and glucuronide conjugates in human biological fluids as a result of coffee consumption. Org. Biomol. Chem..

[25] Fujiwara S., Sakurai S., Sugimoto I., Awata N. (2008). Absorption and metabolism of γ-oryzanol in rats. Chem. Pharm. Bull..

[26] De Karina I.T., Jauregui O., Castellote A.I., Lamuelaraventós R.M., Covas M.I. (2006). Rapid high-performance liquid chromatography-electrospray ionization tandem mass spectrometry method for qualitative and quantitative analysis of virgin olive oil phenolic metabolites in human low-density lipoproteins. J. Chromatogr. A.

[27] Khymenets O., Joglar J., Clapés P., Parella T., Covas M. (2010). Biocatalyzed synthesis and structural characterization of monoglucuronides of hydroxytyrosol, tyrosol, homovanillic alcohol, and 3-(4′-hydroxyphenyl)propanol. Adv. Synth. Catal..

[28] Thibaut R., Debrauwer L., Rao D., Cravedi J.P. (1998). Characterization of biliary metabolites of 4-n-nonylphenol in rainbow trout (Oncorhynchus mykiss). Xenobiotica.

[29] Kajimoto T., Hidaka M., Shoyama K., Nohara T. (1989). Iridoids from *Scrophularia ningpoensis*. Phytochemistry.

[30] Liu X.F., Ouyang S.S., Yu B., Liu B., Liu Y.B., Huang K., Gong J.Y., Zheng S.Y., Li Z.H., Li H.L. (2010). PharmMapper server: A web server for potential drug target identification using pharmacophore mapping approach. Nucleic Acids Res..

[31] Wang X., Pan C.X., Gong J.Y., Liu X.F., Li H.L. (2016). Enhancing the enrichment of pharmacophore-based target prediction for the polypharmacological profiles of drugs. J. Chem. Inf. Model..

[32] Wang X., Shen Y.H., Wang S.W., Li S.L., Zhang W.L., Liu X.F., Lai L.H., Pei J.F., Li H.L. (2017). PharmMapper 2017 update: A web server for potential drug target identification with a comprehensive target pharmacophore database. Nucleic Acids Res..

