# Supplementary material for: Investigation of the In Vivo Metabolism of Sibirioside A and Angoroside C in Rats by HPLC-ESI-IT-TOF-MSn
Source: Molecules. 2018 Oct 19;23(10):2702. doi: 10.3390/molecules23102702 (PMC6222638; doi:10.3390/molecules23102702)
Supplement: Supplementary file 1 [file molecules-23-02702-s001.pdf]

Supplementary data

Investigation of the in vivo metabolism of sibirioside A and angoroside C in rats by HPLC-ESI-IT-TOF-MS^n

Yi-Fan Zhang, Li-Jia Liu, Feng Xu, Ming-Ying Shang*, Guang-Xue Liu, Shao-Qing Cai

Division of Pharmacognosy, School of Pharmaceutical Sciences, Peking University Health Science Center, No.38, Xueyuan Road, Beijing 100191, China; zhangyf0911@163.com (Y.-F.Z.); lily199109231@163.com (L.-J.L.); xufeng_pharm@163.com (F.X.); guangxl@bjmu.edu.cn (G.-X.L.); sqcai@bjmu.edu.cn (S.-Q.C.)

* Correspondence: myshang@bjmu.edu.cn (M.-Y.S.); Tel.: +86-10-8280-2534

Figure S1. The ESI-IT-TOF MS^n spectra of angoroside C (A) and sibirioside A (B).

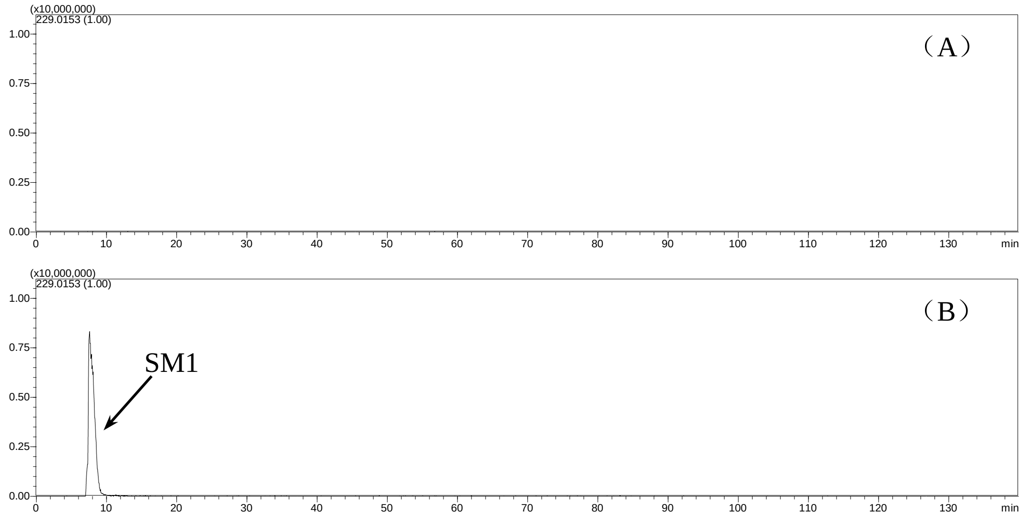

Figure S2. The negative ion mode EIC of SM1 in feces samples (A) blank group; (B) sibirioside A group.

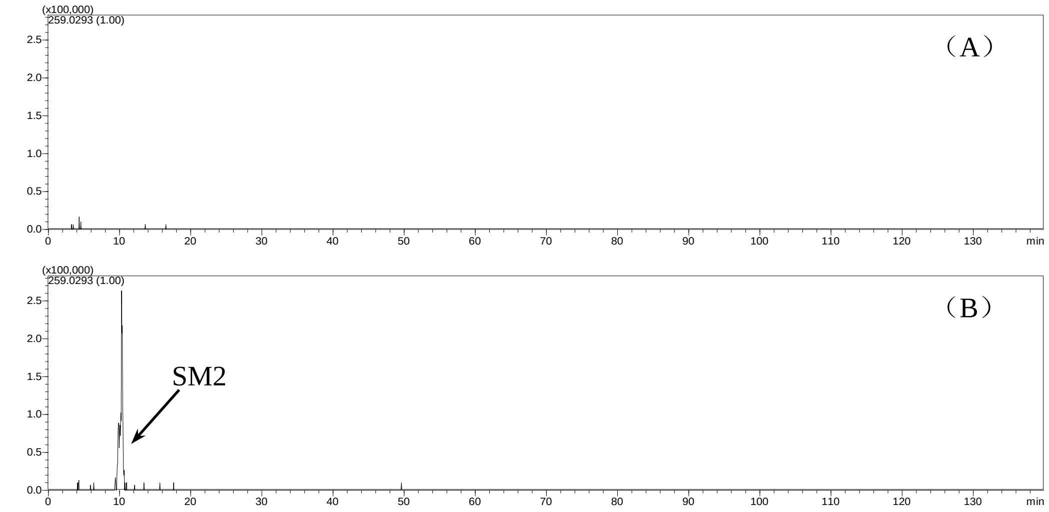

Figure S3. The negative ion mode EIC of SM2 in feces samples (A) blank group; (B) sibirioside A group.

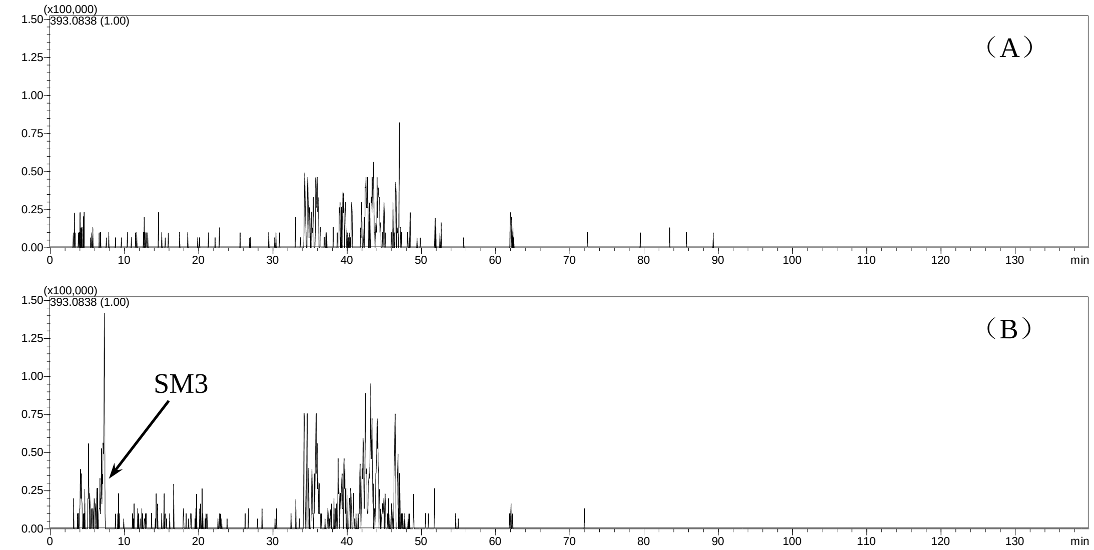

Figure S4. The negative ion mode EIC of SM3 in feces samples (A) blank group; (B) sibirioside A group.

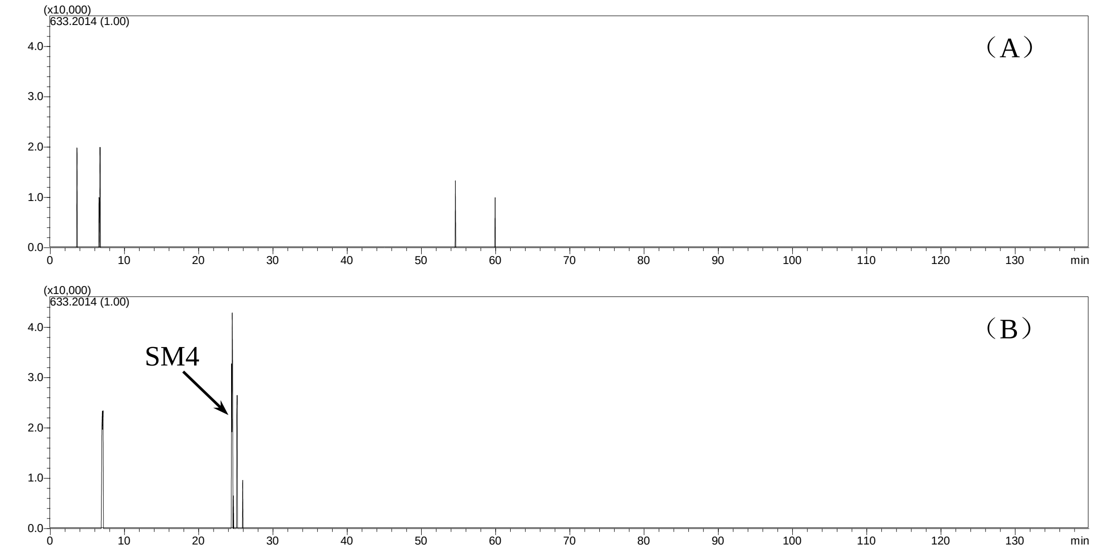

Figure S5. The negative ion mode EIC of SM4 in stomach samples (A) blank group; (B) sibirioside A group.

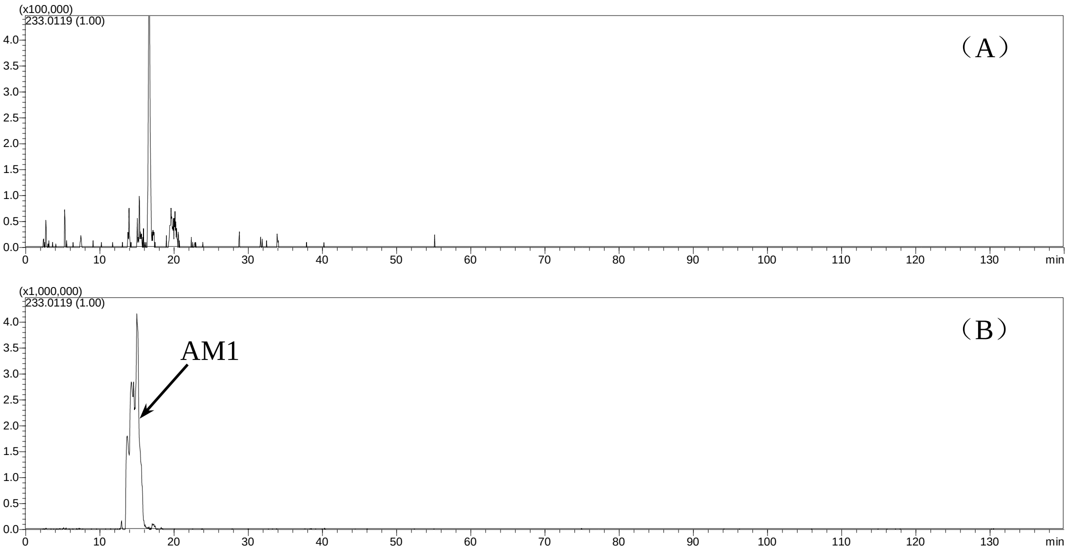

Figure S6. The negative ion mode EIC of AM1 in urine samples (A) blank group; (B) angoroside C group.

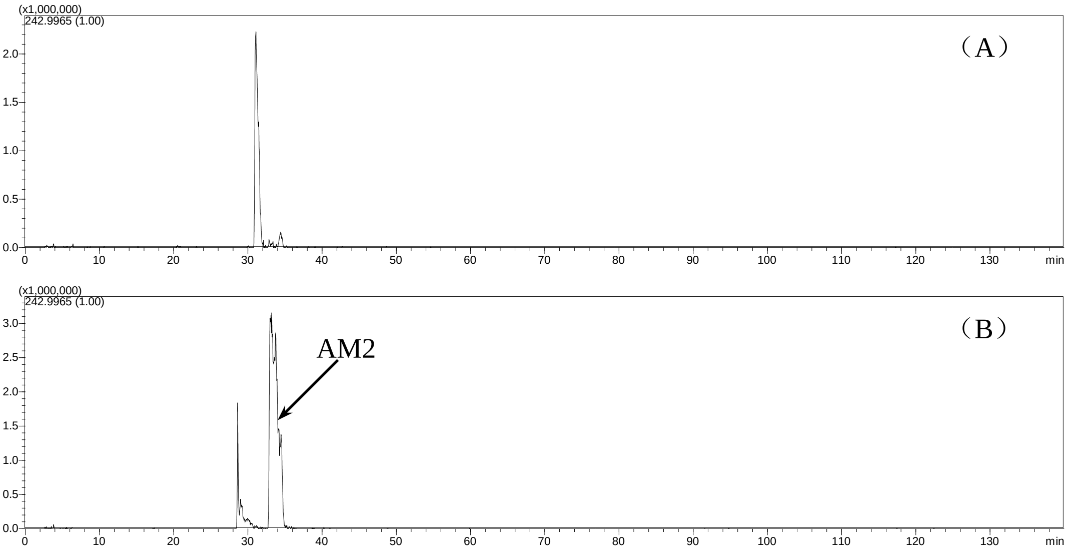

Figure S7. The negative ion mode EIC of AM2 in urine samples (A) blank group; (B) angoroside C group.

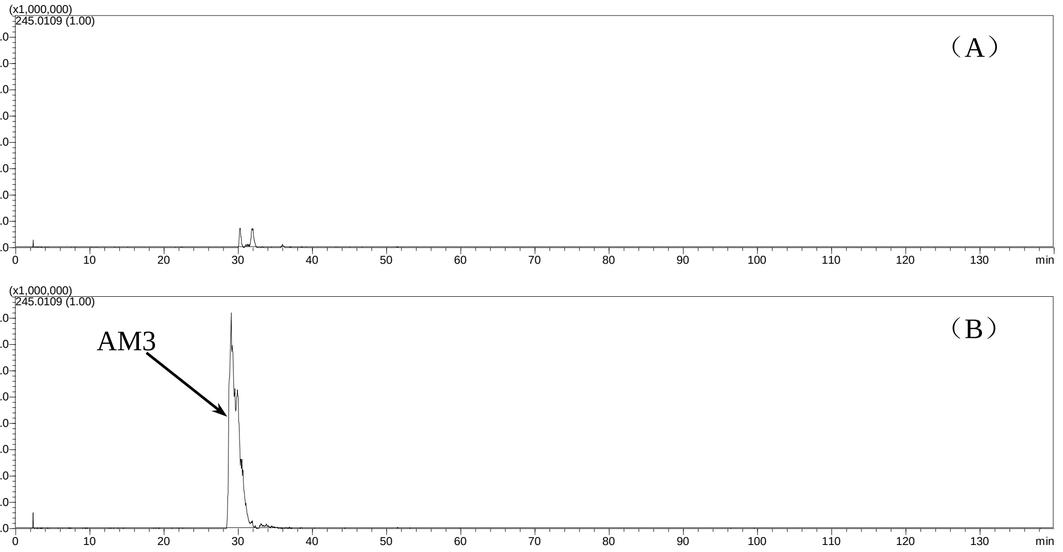

Figure S8. The negative ion mode EIC of AM3 in urine samples (A) blank group; (B) angoroside C group.

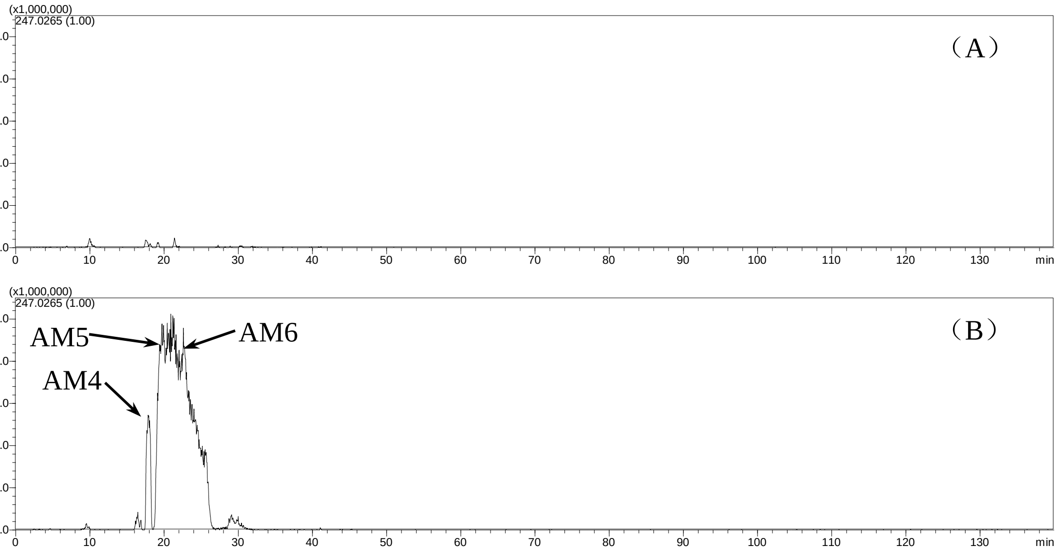

Figure S9. The negative ion mode EIC of AM4, AM5, AM6 in urine samples (A) blank group; (B) angoroside C group.

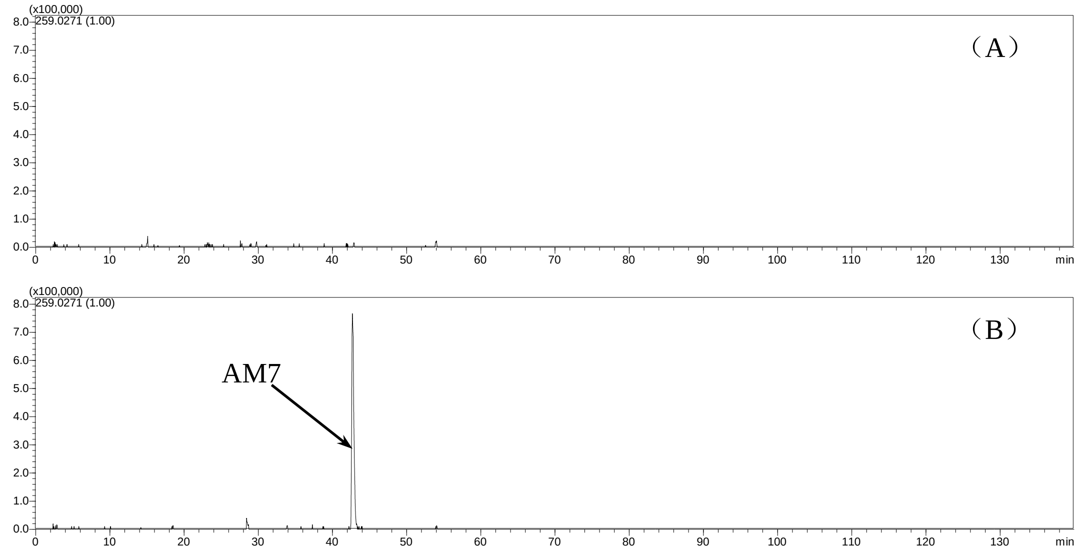

Figure S10. The negative ion mode EIC of AM7 in urine samples (A) blank group; (B) angoroside C group.

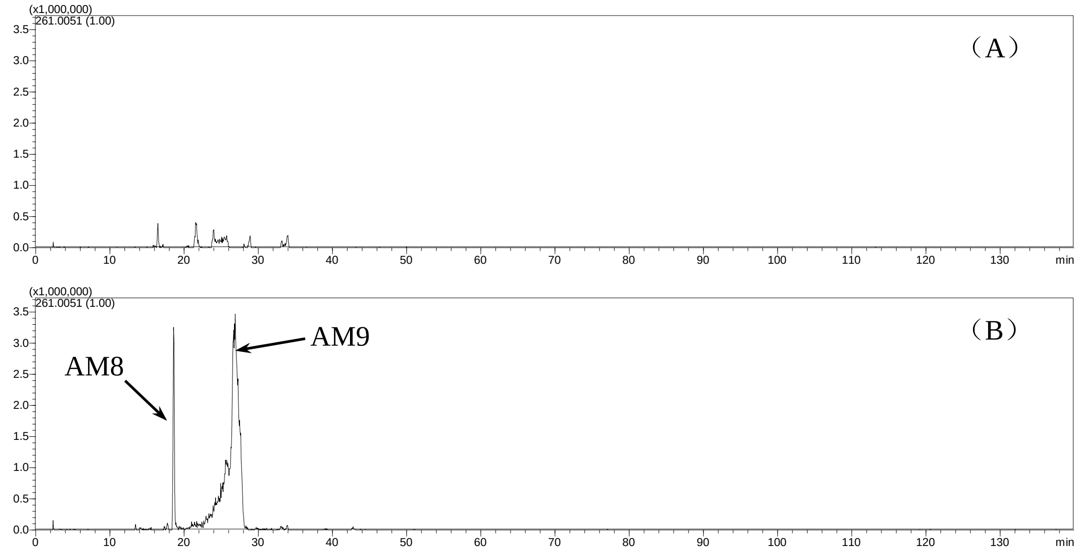

Figure S11. The negative ion mode EIC of AM8, AM9 in urine samples (A) blank group; (B) angoroside C group.

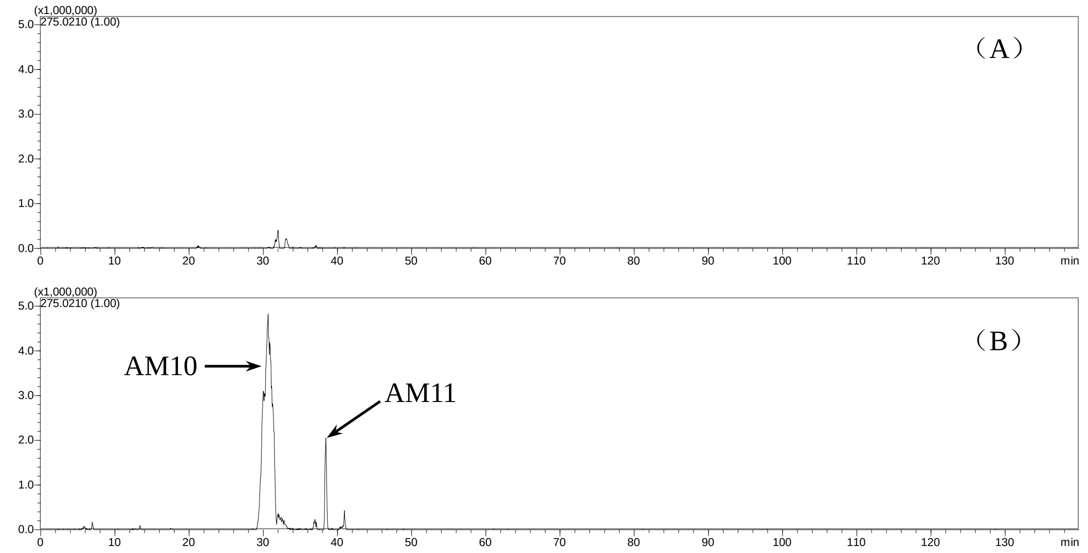

Figure S12. The negative ion mode EIC of AM10, AM11 in urine samples (A) blank group; (B) angoroside C group.

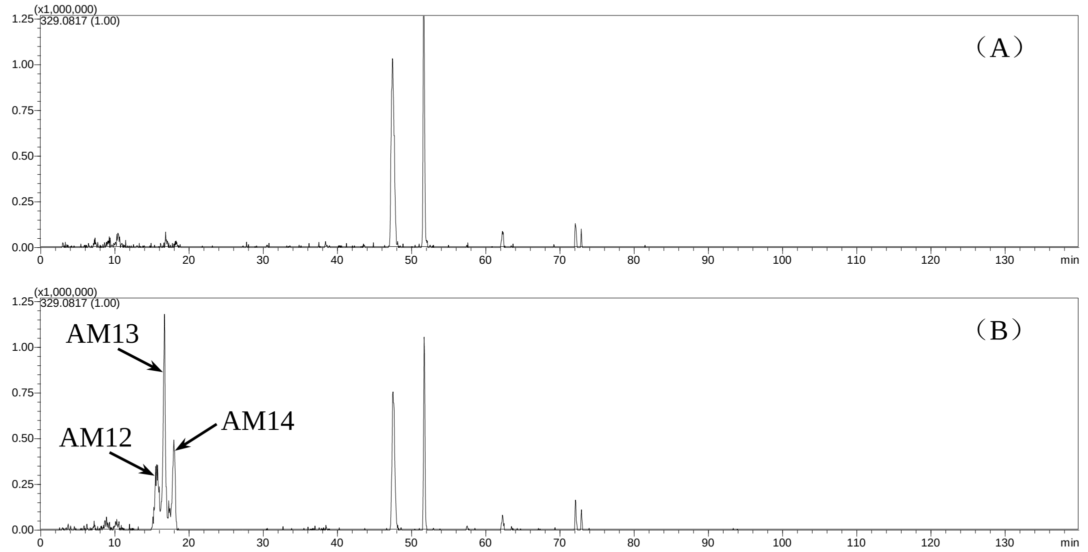

Figure S13. The negative ion mode EIC of AM12, AM13, AM14 in urine samples (A) blank group; (B) angoroside C group.

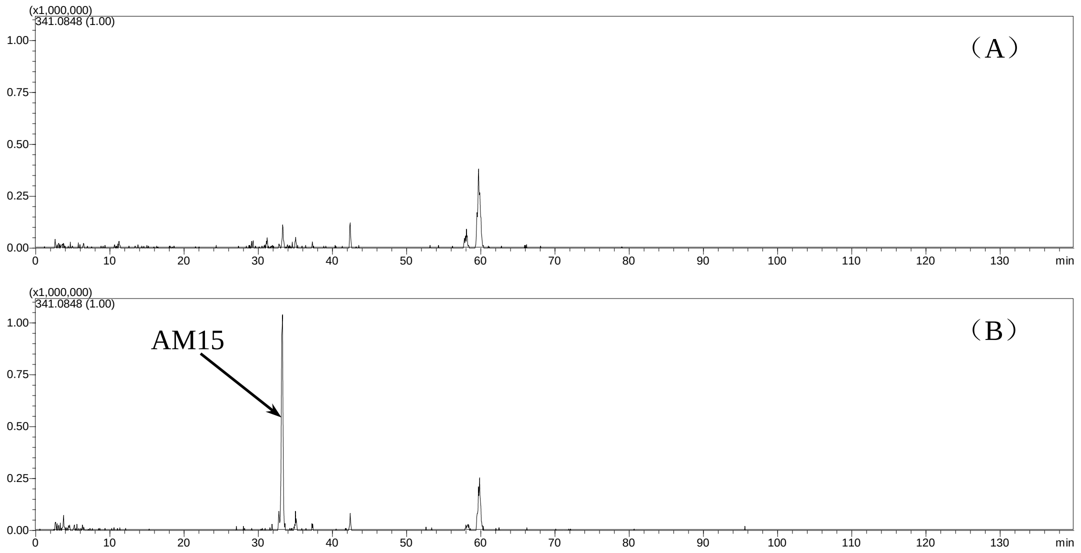

Figure S14. The negative ion mode EIC of AM15 in urine samples (A) blank group; (B) angoroside C group.

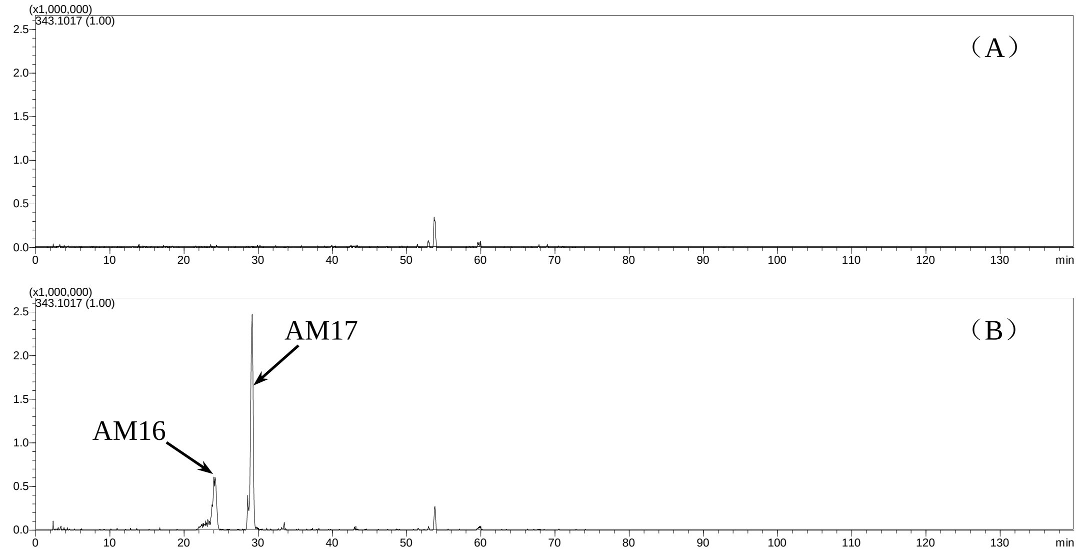

Figure S15. The negative ion mode EIC of AM16, AM17 in urine samples (A) blank group; (B) angoroside C group.

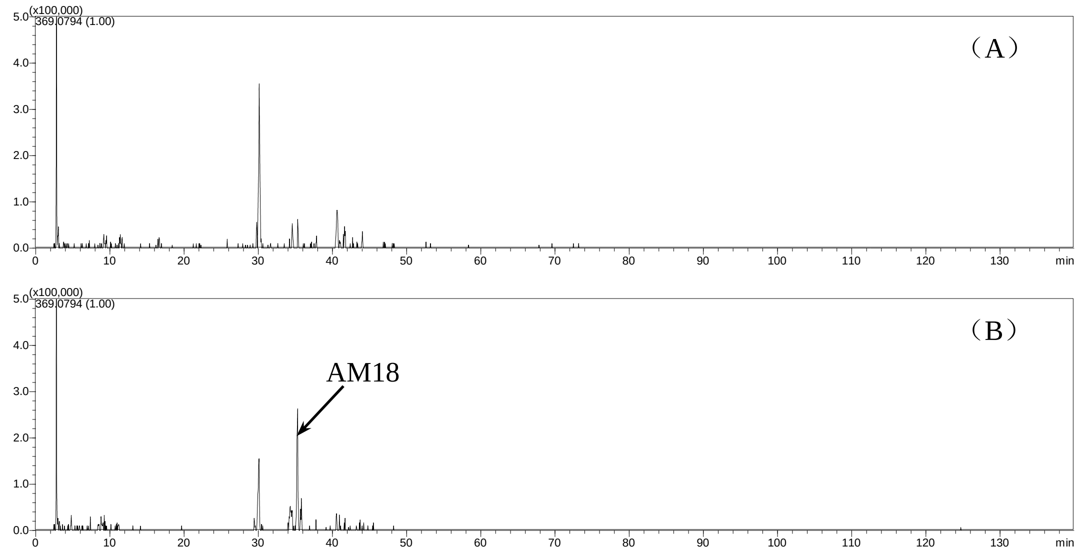

Figure S16. The negative ion mode EIC of AM18 in urine samples (A) blank group; (B) angoroside C group.

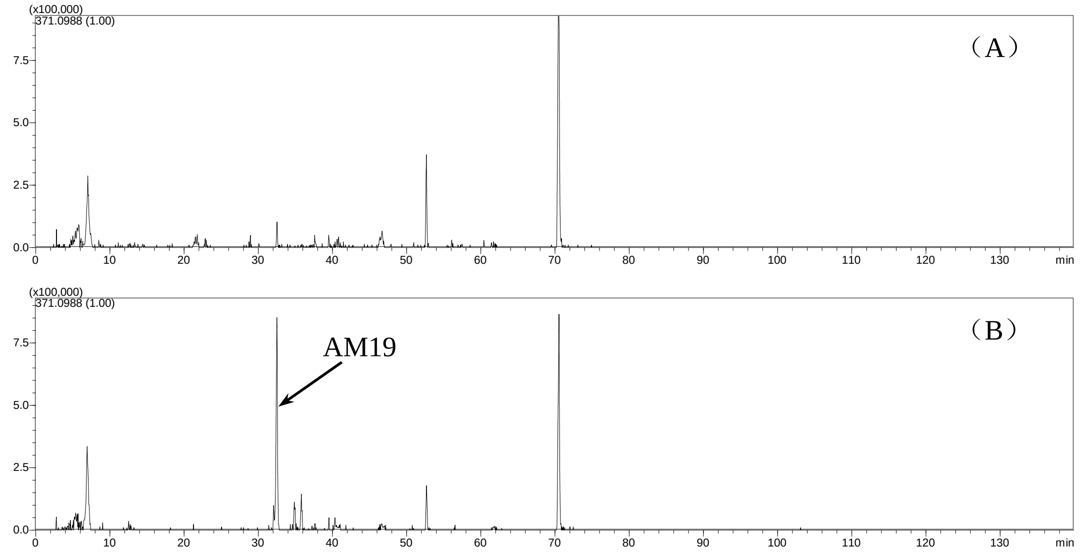

Figure S17. The negative ion mode EIC of AM19 in urine samples (A) blank group; (B) angoroside C group.

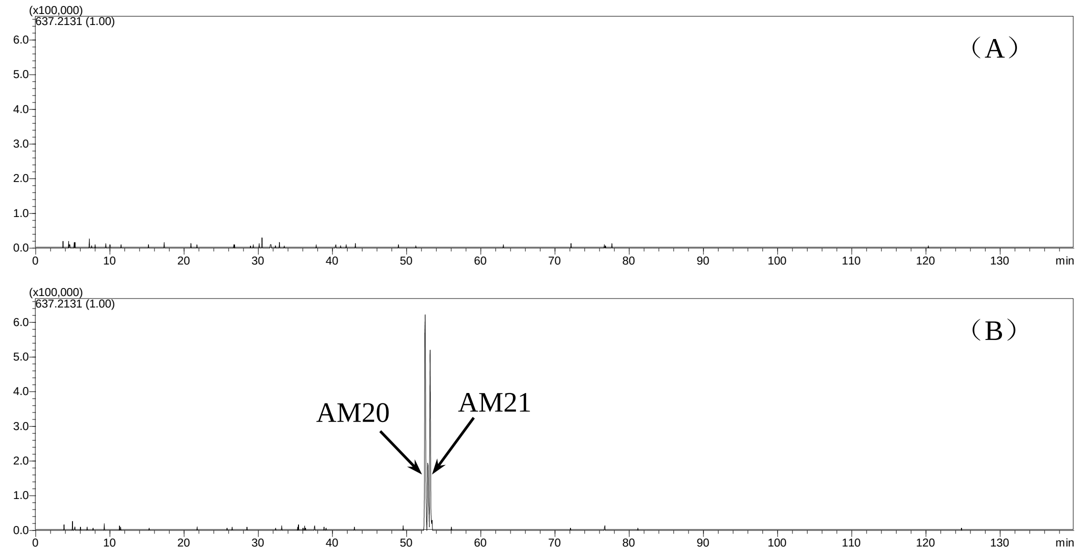

Figure S18. The negative ion mode EIC of AM20, AM21 in urine samples (A) blank group; (B) angoroside C group.

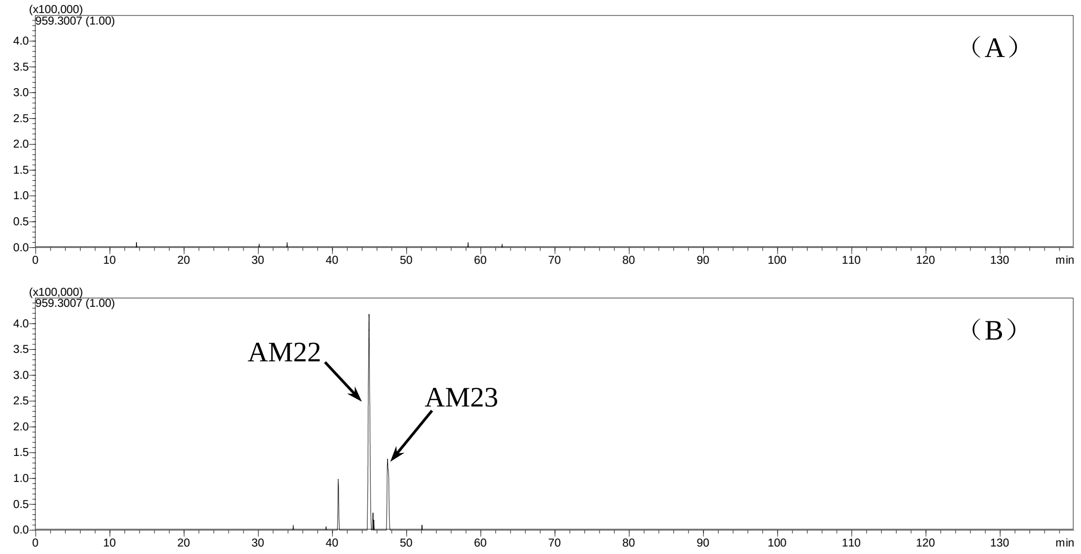

Figure S19. The negative ion mode EIC of AM22, AM23 in urine samples (A) blank group; (B) angoroside C group.

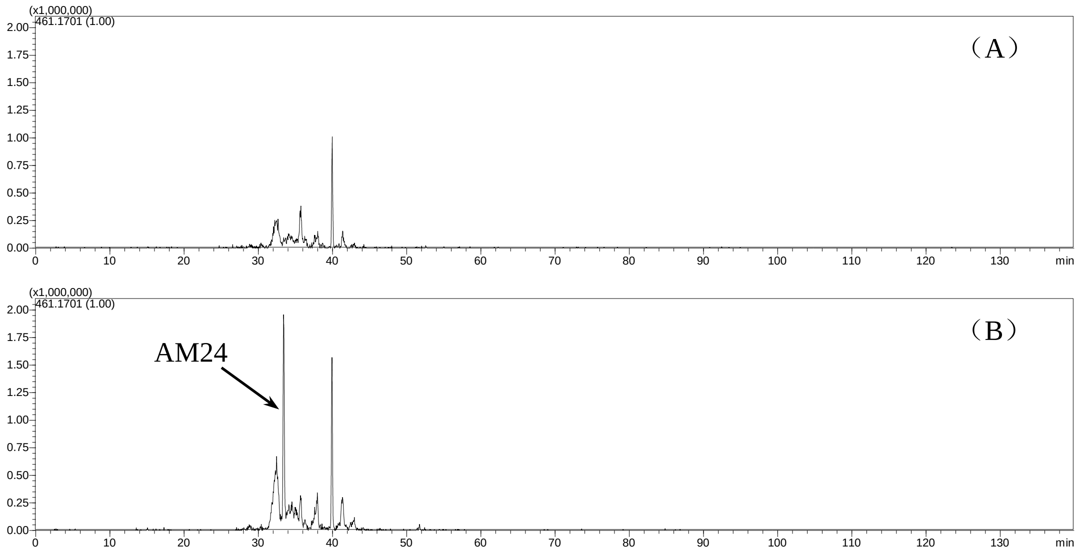

Figure S20. The negative ion mode EIC of AM24 in feces samples (A) blank group; (B) angoroside C group.

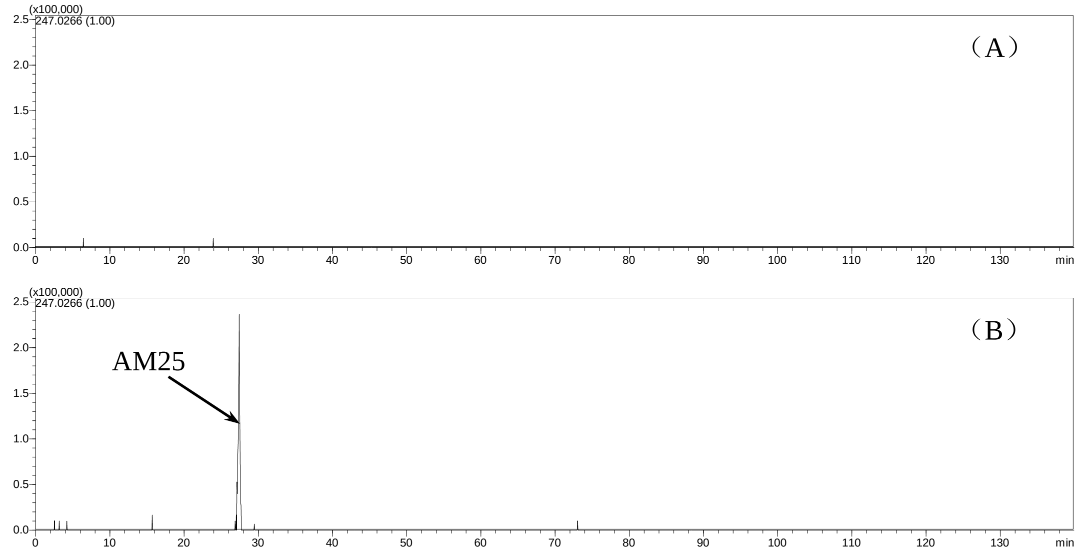

Figure S21. The negative ion mode EIC of AM25 in feces samples (A) blank group; (B) angoroside C group.

Table S1. The predict results of bioactivities for metabolites of sibirioside A and angoroside C.

**No.**	**Potential Targets**	**Potential Activities**	**Target ID**	**Score**
**SM1**	GTPase HRas	Anti-cancer	RASH	4.299
	Angiogenin	Anti myotrophic lateral sclerosis	ANGI	3.904
	Aldose reductase	Anti-diabetes	ALDR	3.821
	Glucose-6-phosphate isomerase	Nonspherocytic hemolytic anemia	G6PI	3.786
	Cathepsin K	Osteogenic activity	CATK	3.744
	Nucleoside diphosphate	Anti-cancer	NDKB	3.730

	kinase B			
	Insulin receptor	Anti-diabetes	INSR	3.666
	Pyruvate kinase PKLR	Anti-hemolytic anemia	KPYR	3.583
	Bifunctional purine biosynthesis protein	Neuroprotective effect	PUR9	3.582
**SM2**	GTPase HRas	Anti-cancer	RASH	5.549
	Nucleoside diphosphate kinase B	Anti-cancer	NDKB	4.580
	Aldose reductase	Anti-diabetes	ALDR	4.404
	Pyruvate kinase PKLR	Anti-hemolytic anemia	KPYR	4.393
	Bifunctional purine biosynthesis protein	Neuroprotective effect	PUR9	4.212
	Insulin receptor	Anti-diabetes	INSR	4.194
	Angiogenin	Anti myotrophic lateral sclerosis	ANGI	3.981
	Fibrinogen gamma chain	Cardiovascular protection	FIBG	3.840
	Glucose-6-phosphate isomerase	Nonspherocytic hemolytic anemia	G6PI	3.837
	Hepatocyte growth factor receptor	Anti-cancer	MET	3.778
	Cathepsin K	Osteogenic activity	CATK	3.763
**SM3**	Pyruvate kinase PKLR	Anti-hemolytic anemia	KPYR	4.373
	Tyrosine-protein kinase BTK	Anti-chronic leukemia	BTK	4.288
	Coagulation factor VII	Anti-coagulopathy	FA7	4.144
	Insulin receptor	Anti-diabetes	INSR	4.139
	Aldose reductase	Anti-diabetes	ALDR	3.936
	GTPase HRas	Anti-cancer	RASH	3.903
	Bifunctional purine biosynthesis protein	Neuroprotective effect	PUR9	3.885
	Glucose-6-phosphate isomerase	Nonspherocytic hemolytic anemia	G6PI	3.861
	Cathepsin K	Osteogenic activity	CATK	3.835
**AM2**	Nucleoside diphosphate kinase B	Anti-cancer	NDKB	4.308
	GTPase HRas	Anti-cancer	RASH	4.219
	Coagulation factor VII	Anti-coagulopathy	FA7	4.059
	Bifunctional purine biosynthesis protein	Neuroprotective effect	PUR9	4.152
	Insulin receptor	Anti-diabetes	INSR	4.127
	Pyruvate kinase PKLR	Anti-hemolytic anemia	KPYR	3.752
	Carbonic anhydrase 2	Osteogenic activity	CAH2	3.692
	Angiogenin	Anti-myotrophic lateral sclerosis	ANGI	3.616

	Tyrosine-protein kinase BTK	Anti-chronic leukemia	BTK	3.613
	Angiotensin-converting enzyme	Cerebral protection	ACE	3.533
**AM4**	Nucleoside diphosphate kinase B	Anti-cancer	NDKB	4.351
	Insulin receptor	Anti-diabetes	INSR	4.096
	Angiogenin	Anti myotrophic lateral sclerosis	ANGI	3.915
	Dual specificity mitogen-activated protein kinase kinase 1	Cardiovascular protection	MP2K1	3.853
	Angiotensin-converting enzyme	Cerebral protection	ACE	3.815
	Pyruvate kinase PKLR	Anti-hemolytic anemia	KPYR	3.780
	Carbonic anhydrase 2	Osteogenic activity	CAH2	3.714
	GTPase HRas	Anti-cancer	RASH	3.714
	Coagulation factor VII	Anti-coagulopathy	FA7	3.704
	Serum albumin		ALBU	3.518
**AM5**	Angiogenin	Anti-myotrophic lateral sclerosis	ANGI	4.076
	Carbonic anhydrase 2	Osteogenic activity	CAH2	3.878
	Nucleoside diphosphate kinase B	Anti-cancer	NDKB	3.837
	GTPase HRas	Anti-cancer	RASH	3.743
	Bifunctional purine biosynthesis protein	Neuroprotective effect	PUR9	3.706
	Prothrombin	Cerebrovascular protection	THRB	3.683
	Dual specificity mitogen-activated protein kinase kinase 1	Cardiovascular protection	MP2K1	3.651
	Pyruvate kinase PKLR	Anti-hemolytic anemia	KPYR	3.617
	Tyrosine-protein kinase BTK	Anti chronic leukemia	BTK	3.611
	Insulin receptor	Anti-diabetes	INSR	3.600
**AM6**	Pyruvate kinase PKLR	Anti-hemolytic anemia	KPYR	4.501
	Insulin receptor	Anti-diabetes	INSR	4.321
	Coagulation factor VII	Anti-coagulopathy	FA7	4.104
	Carbonic anhydrase 2	Osteogenic activity	CAH2	3.810
	Angiotensin-converting enzyme	Cerebral protection	ACE	3.763
	Dual specificity mitogen-activated protein kinase kinase 1	Cardiovascular protection	MP2K1	3.739

	Glucose-6-phosphate 1-dehydrogenase	Nonspherocytic hemolytic anemia	G6PD	3.649
**AM8**	Nucleoside diphosphate kinase B	Anti-cancer	NDKB	4.434
	Tyrosine-protein kinase BTK	Anti-chronic leukemia	BTK	4.410
	Angiogenin	Anti-myotrophic lateral sclerosis	ANGI	4.246
	Pyruvate kinase PKLR	Anti-hemolytic anemia	KPYR	4.229
	GTPase HRas	Anti-cancer	RASH	4.077
	Inositol monophosphatase 1	Anti-manic and anti-depressant	IMPA1	3.924
	Carbonic anhydrase 2	Osteogenic activity	CAH2	3.861
	Glucose-6-phosphate isomerase	Nonspherocytic hemolytic anemia	G6PI	3.844
	Fibrinogen gamma chain	Cardiovascular protection	FIBG	3.833
	Bifunctional purine biosynthesis protein	Neuroprotective effect	PUR9	3.704
**AM9**	Nucleoside diphosphate kinase B	Anti-cancer	NDKB	4.397
	GTPase HRas	Anti-cancer	RASH	4.105
	Angiogenin	Anti myotrophic lateral sclerosis	ANGI	4.095
	Coagulation factor VII	Anti-coagulopathy	FA7	4.077
	Bifunctional purine biosynthesis protein	Neuroprotective effect	PUR9	4.046
	Tyrosine-protein kinase BTK	Anti-chronic leukemia	BTK	3.994
	Glucose-6-phosphate 1-dehydrogenase	Nonspherocytic hemolytic anemia	G6PD	3.905
	Carbonic anhydrase 2	Osteogenic activity	CAH2	3.810
	Pyruvate kinase PKLR	Anti-hemolytic anemia	KPYR	3.797
	Insulin receptor	Anti-diabetes	INSR	3.742
	Fibrinogen gamma chain	Cardiovascular protection	FIBG	3.664
	Mitogen-activated protein kinase 10	Anti-epileptic encephalopathy	MK10	3.653
	Angiotensin-converting enzyme	Cerebral protection	ACE	3.629
**AM10**	Angiogenin	Anti-myotrophic lateral sclerosis	ANGI	4.628
	GTPase HRas	Anti-cancer	RASH	4.482
	Tyrosine-protein kinase BTK	Anti-chronic leukemia	BTK	4.445
	Pyruvate kinase PKLR	Anti-hemolytic anemia	KPYR	4.253

	Nucleoside diphosphate kinase B	Anti-cancer	NDKB	4.083
	Coagulation factor VII	Anti-coagulopathy	FA7	3.995
	Glucose-6-phosphate isomerase	Nonspherocytic hemolytic anemia	G6PI	3.871
	Carbonic anhydrase 2	Osteogenic activity	CAH2	3.832
	Insulin receptor	Anti-diabetes	INSR	3.813
**AM11**	Nucleoside diphosphate kinase B	Anti-cancer	NDKB	4.617
	Angiogenin	Anti-myotrophic lateral sclerosis	ANGI	4.398
	GTPase HRas	Anti-cancer	RASH	4.269
	Pyruvate kinase PKLR	Anti-hemolytic anemia	KPYR	4.248
	Tyrosine-protein kinase BTK	Anti-chronic leukemia	BTK	4.050
	Glucose-6-phosphate isomerase	Nonspherocytic hemolytic anemia	G6PI	3.872
	cAMP-specific 3',5'-cyclic phosphodiesterase 4D	Anti-stroke	PDE4D	3.848
	Carbonic anhydrase 2	Osteogenic activity	CAH2	3.842
	Insulin receptor	Anti-diabetes	INSR	3.773
**AM12**	GTPase HRas	Anti-cancer	RASH	4.089
	Pyruvate kinase PKLR	Anti-hemolytic anemia	KPYR	3.795
	Glucose-6-phosphate 1-dehydrogenase	Nonspherocytic hemolytic anemia	G6PD	3.782
	Coagulation factor VII	Anti-coagulopathy	FA7	3.775
	Inositol monophosphatase 1	Anti-manic and anti-depressant	IMPA1	3.742
	Aldose reductase	Anti-diabetes	ALDR	3.708
	Ornithine aminotransferase	Anti-hyperornithinemia	OAT	3.699
	Tyrosine-protein kinase Lck	Anti-leukemias	LCK	3.599
	Dual specificity mitogen-activated protein kinase kinase 1	Cardiovascular protection	MP2K1	3.529
	Glucose-6-phosphate isomerase	Nonspherocytic hemolytic anemia	G6PI	3.496
	Serine--pyruvate aminotransferase	Anti-hyperoxaluria	SPYA	3.434
	Insulin receptor	Anti-diabetes	INSR	3.432
	Mitogen-activated protein kinase 10	Anti-epileptic encephalopathy	MK10	3.418
	Angiogenin	Anti-myotrophic lateral	ANGI	3.384

		sclerosis		
**AM13**	GTPase HRas	Anti-cancer	RASH	3.889
	Coagulation factor VII	Anti-coagulopathy	FA7	3.591
	Pyruvate kinase PKLR	Anti-hemolytic anemia	KPYR	3.468
	Glucose-6-phosphate 1-dehydrogenase	Nonspherocytic hemolytic anemia	G6PD	3.408
	Angiogenin	Anti-myotrophic lateral sclerosis	ANGI	3.388
	Insulin receptor	Anti-diabetes	INSR	3.323
	Mitogen-activated protein kinase 10	Anti-epileptic encephalopathy	MK10	3.246
	Inositol monophosphatase 1	Anti-manic and anti-depressant	IMPA1	3.156
	Aldose reductase	Anti-diabetes	ALDR	2.992
	Ornithine aminotransferase	Anti-hyperornithinemia	OAT	2.970
	Complement C1s subcomponent	Anti-autoimmune diseases	C1S	2.910
	cAMP-specific 3',5'-cyclic phosphodiesterase 4D	Anti-stroke	PDE4D	2.906
	Catalase	Anti-acatalasia	CATA	2.896
	Serum albumin	Anti-dysalbuminemic hyperthyroxinemia	ALBU	2.887
	Prothrombin	Anti-dysprothrombinemia	THRB	2.884
**AM14**	GTPase HRas	Anti-cancer	RASH	3.770
	Angiogenin	Anti-myotrophic lateral sclerosis	ANGI	3.388
	Ornithine aminotransferase	Anti-hyperornithinemia	OAT	3.338
	Coagulation factor VII	Anti-coagulopathy	FA7	3.316
	Tyrosine-protein kinase BTK	Anti-chronic leukemia	BTK	3.313
	Glucose-6-phosphate 1-dehydrogenase	Nonspherocytic hemolytic anemia	G6PD	3.034
	Aldose reductase	Anti-diabetes	ALDR	2.992
	Mitogen-activated protein kinase 10	Anti-epileptic encephalopathy	MK10	2.955
	Transthyretin	Anti-amyloidosis	TTHY	2.952
	Nucleoside diphosphate kinase B	Anti-cancer	NDKB	2.946
	Carbonic anhydrase 2	Osteogenic activity	CAH2	2.923
	Complement C1s subcomponent	Anti-autoimmune diseases	C1S	2.910
	Tyrosine-protein kinase	Anti-leukemias	LCK	2.868

	Lck			
**AM15**	Insulin receptor	Anti-diabetes	INSR	5.223
	Glucose-6-phosphate isomerase	Nonspherocytic hemolytic anemia	G6PI	4.924
	GTPase HRas	Anti-cancer	RASH	4.901
	Inositol monophosphatase 1	Anti-manic and anti-depressant	IMPA1	4.873
	Mitogen-activated protein kinase 10	Anti-epileptic encephalopathy	MK10	4.676
	Tyrosine-protein kinase Lck	Anti-leukemias	LCK	4.390
	Pyruvate kinase PKLR	Anti-hemolytic anemia	KPYR	4.333
	Tyrosine-protein kinase BTK	Anti-chronic leukemia	BTK	4.156
	Ornithine aminotransferase	Anti-hyperornithinemia	OAT	4.117
	Angiogenin	Anti myotrophic lateral sclerosis	ANGI	4.106
	Coagulation factor VII	Anti-coagulopathy	FA7	4.082
	cAMP-specific 3',5'-cyclic phosphodiesterase 4D	Anti-stroke	PDE4D	4.106
**AM18**	Insulin receptor	Anti-diabetes	INSR	5.187
	Inositol monophosphatase 1	Anti-manic and anti-depressant	IMPA1	4.910
	Tyrosine-protein kinase BTK	Anti-chronic leukemia	BTK	4.874
	GTPase HRas	Anti-cancer	RASH	4.859
	Mitogen-activated protein kinase 10	Anti-epileptic encephalopathy	MK10	4.627
	Coagulation factor VII	Anti-coagulopathy	FA7	4.508
	Glucose-6-phosphate isomerase	Nonspherocytic hemolytic anemia	G6PI	4.497
	Angiotensin-converting enzyme	Cerebral protection	ACE	4.440
	Ornithine aminotransferase	Anti-hyperornithinemia	OAT	4.246
	Angiogenin	Anti-myotrophic lateral sclerosis	ANGI	4.233
**AM19**	Insulin receptor	Anti-diabetes	INSR	5.277
	Inositol monophosphatase 1	Anti-manic and anti-depressant	IMPA1	4.960
	Tyrosine-protein kinase BTK	Anti-chronic leukemia	BTK	4.865
	GTPase HRas	Anti-cancer	RASH	4.965

	Mitogen-activated protein kinase 10	Anti-epileptic encephalopathy	MK10	4.540
	Pyruvate kinase PKLR	Anti-hemolytic anemia	KPYR	4.484
	Coagulation factor VII	Anti-coagulopathy	FA7	4.429
	Angiogenin	Anti-myotrophic lateral sclerosis	ANGI	4.258
	Glucose-6-phosphate isomerase	Nonspherocytic hemolytic anemia	G6PI	4.168
	Aldose reductase	Anti-diabetes	ALDR	4.023
**AM24**	Tyrosine-protein kinase BTK	Anti-chronic leukemia	BTK	3.786
	Insulin receptor	Anti-diabetes	INSR	3.753
	GTPase HRas	Anti-cancer	RASH	3.499
	Aldose reductase	Anti-diabetes	ALDR	3.210
	Mitogen-activated protein kinase 10	Anti-epileptic encephalopathy	MK10	3.161
	Carbonic anhydrase 2	Osteogenic activity	CAH2	3.108
	Prothrombin	Cerebrovascular protection	THRB	3.071
	Coagulation factor VII	Anti-coagulopathy	FA7	3.053
	Glucose-6-phosphate 1-dehydrogenase	Nonspherocytic hemolytic anemia	G6PD	2.988
**AM25**	GTPase HRas	Anti-cancer	RASH	4.556
	Bifunctional purine biosynthesis protein	Neuroprotective effect	PUR9	4.465
	Insulin receptor	Anti-diabetes	INSR	4.264
	Tyrosine-protein kinase BTK	Anti-chronic leukemia	BTK	4.247
	Pyruvate kinase PKLR	Anti-hemolytic anemia	KPYR	4.161
	Nucleoside diphosphate kinase B	Anti-cancer	NDKB	4.144
	Angiotensin-converting enzyme	Cerebral protection	ACE	3.892
	Inosine-5'-monophosphate dehydrogenase 1	retinitis pigmentosa	IMDH1	3.771
	Glucose-6-phosphate 1-dehydrogenase	Nonspherocytic hemolytic anemia	G6PD	3.749
	Signal transducer and activator of transcription 1-alpha/beta	Immune related	STAT1	3.746
